# The Tanggula Mountains enhance population divergence in *Carex moorcroftii*: a dominant sedge on the Qinghai-Tibetan Plateau

**DOI:** 10.1038/s41598-018-21129-y

**Published:** 2018-02-09

**Authors:** Wensheng Liu, Yao Zhao, Danhui Qi, Jianling You, Yin Zhou, Zhiping Song

**Affiliations:** 1 Ministry of Education Key Laboratory for Biodiversity Science and Ecological Engineering, Institute of Biodiversity Science, Fudan University, Shanghai, 200433 China; 2 Central South University of Forestry and Techonology, Changsha, 410018 China; 3 Southwest Forestry University, Kunming, 650224 China

## Abstract

High-altitude mountains are often geographic barriers to gene flow and play important roles in shaping population divergence. The central Qinghai-Tibetan Plateau (QTP) stands the location of the Tanggula Mountains (TM). We use the TM as a case, using *Carex moorcroftii*, a dominant species on the QTP to test the effects of geographic barriers on plant population divergence. We sampled 18 *C. moorcroftii* populations along a north-south transect crossing the TM to investigate the correlations of genetic variation and morphological traits with climate variables. The results showed this species holds high genetic diversity (*H*_e_ = 0.58) and the surveyed populations can be genetically clustered into two groups: populations from the north face of TM, and the other from the south. Gene flow between populations within groups is higher than those between groups. The traits, number and mass of seeds, mass of root and infructescence significantly varied among populations. Mantel-tests detected a weak but significantly positive correlation between genetic and geographic (*R*^2^ = 0.107, *p* = 0.032) and climatic distance (*R*^2^ = 0.162, *p* = 0.005), indicating both isolation by distance and isolation by environment. These findings together suggest high-altitude mountains of TM interrupt habitat continuity, result in distinct climatic conditions on both sides, increasing population divergence of plant species.

## Introduction

High-altitude mountains, due to their physical characteristics, would alter the environment around them, leading to increased habitat heterogeneity (might result in local adaptation, then could be considered as environmental barrier) and possible geographical barriers to gene flow^1,2^. Both habitat heterogeneity and geographical barriers may directly affect evolutionary processes and genetic diversity of species^3^. Specifically, geographical barriers can limit dispersal ability leading to interrupted gene flow and increased population divergence, which in extreme cases may result in reproductive isolation^4^. However, in the absence of a geographical barrier, genetic divergence may still occur, primarily due to the effect of isolation by distance (IBD), which acts through genetic drift and dispersal limitation^5^. Alternatively, isolation by environment (IBE) is expected under the action of natural selection, which is influenced by habitat divergence^6^. Gene flow may counteract the effects of IBE and increase intra-population genetic variations and inter-population genetic connectivity^6,7^. This may enhance species persistence and adaptive ability under climate change^6^. For instance, gene flow from populations pre-adapted to warmer environmental conditions is expected to increase the recipient populations’ ability to adapt to increasing temperatures^8^. In a Swedish population of *Pinus sylvestris*, Nilsson^9^ found that the progenies sired by long-distance pollen had higher freezing resistance compared with that produced by local pollen. A comparison between pine and birch also showed that long-distance gene flow mediated by pollen and/or seed along the climatic gradient might moderately promote populations’ adaptation to climate warming^10^.

Morphological characters or genetic variation can be altered in plants in response to different environmental conditions. Revealing how environmental heterogeneity influences genetic variation and plant performance is of paramount significance in understanding the capacity of plant to respond to global climate change in real time^11^. Intraspecific variation might be associated with environmental variables that fall along a latitudinal gradient, and the effects of these variable factors on population dynamics is increasingly recognized^12,13^. Uncovering the morphological and genetic variations of plant populations associated with particular environments along latitudinal gradients is an effective method to answer this question^12^.

The Qinghai-Tibetan Plateau (QTP) is the highest place in the world, making it one of the most vulnerable places to global climate change^14,15^. With the uplift of the Himalaya, precipitation and air temperature increase from northwest to southeast on this plateau^16^. The snow-covered, high-altitudinal mountains and deep valleys affiliated with the QTP characterize the environmental gradients that act as geographic barriers to gene flow between populations at the regional scale. For instance, the Tanggula Mountains (TM) lie east to west on the central QTP with a mean altitude over 5,000 m a.s.l. The south side of the TM has more precipitation than the north, resulting in differences in the soil and ambient humidity^17,18^. Such environmental differences contribute to habitat heterogeneity, which can have strong effects on community assemblage by influencing the local populations. Additionally, the ridge of TM is always covered by snow or glacier, forming a vegetative gap and disrupting connections between populations on both sides^15,19–21^. These features make the TM an ideal model to test the effects of geographic barriers on population divergence, since the effects of the TM on the evolutionary process of plant species dispersal on this mountain has not been fully investigated.

To investigate the effects of the TM on population divergence, *Carex moorcroftii* is an ideal study system because it is a widespread dominant species of alpine steppe and meadow on the QTP. Additionally, It is a wind-pollination and gravity seed-dispersal species, which can clonally grow and propagate by stolons below ground. The distribution of *C. moorcroftii* crosses diverse climatic gradients on the QTP, especially crossing the TM. We hypothesize that (1) the north-south connection between *C. moorcroftii* populations is interrupted by the physical barrier of the TM; and (2) both morphological and genetic variations of *C. moorcroftii* will correspond to the different habitats found on either side of the TM. To test these hypotheses, we sampled 18 *C. moorcroftii* populations along a north-south transect crossing the TM, estimated morphological and genetic variations of these populations and correlated these variations with environment gradients. We would specifically answer the follow questions: (1) Do morphological and genetic variations of populations correlate with environmental changes? (2) Does the TM disrupt gene flow and enhance population divergence between the north and south sides? Such knowledge is useful to assess the ability of long distance dispersal of *C. moorcroftii*, which is important for the alpine plants to track the distribution shift caused by climate changes.

## Results

### Ecological niche modeling and habitat heterogenity

18 populations were sampled (Table 1). The ENM (Ecological Niche modeling) model showed high accuracy with a training AUC of 0.966 and a testing AUC of 0.962. The number of false negatives (omission error, fraction of test data omitted at logistic threshold for maximum test sensitivity plus specificity) was as high as 1.42%. The highest contributing variable was *MTWaQ* (Bio10), with a contribution of 34.5%. Additionally, *MTWaM* (Bio5) and Temperature Seasonality (standard deviation*100) (*TS*, Bio4) were the next most important contributors to the distribution of *C. moorcroftii* (Fig. S1; Table S1), which had a contribution of 22.6% and 16.6%. The top three variables contributed 73.67% in the model’s explanatory ability in total. The importance of these variables was also demonstrated in the jackknife evaluation of the training gain, gain test and AUC test, as these variables used individually in the model still contributed the most (Fig. S2).

**Table 1:** Latitude, longitude, altitude and individual density of *Carex moorcroftii* populations sampled along a latitudinal transect on the Qinghai-Tibetan Plateau.

Population	Code	Latitude (N)	Longitude (E)	Altitude (m)	Shoot density (shoots m^−2^)	Individual number	Flowering shoot density (N m^−2^)	Flowering shoot (% of total)
Xidatan	P1	35°45′	94°19′	4123	44.40 ± 31.24	27	2.00 ± 2.11	10.34 ± 14.34
Budongquan	P2	35°31′	93°54′	4612	95.20 ± 63.86	28	5.20 ± 4.64	5.56 ± 6.18
Suonandajie	P3	35°31′	93°45′	4568	71.20 ± 31.04	45	9.60 ± 4.56	13.24 ± 2.17
Wudaoliang	P4	35°14′	93°05′	4672	151.20 ± 131.74	29	15.20 ± 10.35	25.51 ± 22.36
Erdaogou	P5	34°49′	92°56′	4644	76.80 ± 56.37	31	4.00 ± 4.62	4.82 ± 6.25
Tuotuohe station	P6	34°37′	92°47′	4725	25.60 ± 22.91	28	4.80 ± 3.35	21.11 ± 18.32
Tuotuohe	P7	34°16′	92°29′	4571	53.60 ± 48.16	39	6.80 ± 11.16	10.81 ± 17.01
Yanshiping	P8	33°43′	92°05′	4684	349.20 ± 275.34	47	5.20 ± 5.67	6.30 ± 12.56
Tang 1	P9	32°45′	91°53′	5026	22.00 ± 15.34	35	8.00 ± 3.58	46.35 ± 30.89
Tang 2	P10	32°31′	91°50′	4930	52.00 ± 41.90	40	7.56 ± 9.68	17.62 ± 26.02
Tang 3	P11	32°24′	91°44′	4814	44.00 ± 29.21	25	5.71 ± 6.87	10.41 ± 10.50
Anduo	P12	32°06′	91°41′	4719	77.71 ± 37.66	30	13.14 ± 17.70	13.47 ± 12.56
Naqu	P13	31°39′	91°46′	4575	206.40 ± 119.35	47	20.80 ± 8.67	11.36 ± 3.20
Luomaozhen	P14	31°20′	91°54′	4535	98.00 ± 189.93	44	12.00 ± 25.02	13.00 ± 21.13
Yangbajin	P15	30°18′	90°48′	4332	50.91 ± 38.04	27	0.00 ± 0.00	0.00 ± 0.00
Everest 4900	P16	28°13′	86°49′	4918	92.80 ± 52.18	40	9.60 ± 6.69	9.80 ± 2.52
Yela mountain	P17	30°09′	97°19′	4658	195.20 ± 81.46	40	24.00 ± 8.94	12.98 ± 3.37
Chuanzangxian	P18	30°41′	97°15′	4048	163.20 ± 61.41	36	18.40 ± 7.27	12.29 ± 5.96

PCA results of all 19 climatic variables showed that the loadings for PC1, PC2 and PC3 were 11.33, 3.25 and 1.95, and the percentage of variation captured ranging from 59.7% to 17.1% and to 10.3%, respectively. Variables whose absolute values of factor loading above 0.85 for PC1 were Mean Temperature of Coldest Quarter (*MTCQ*, Bio11**)**, Mean Temperature of Driest Quarter (*MTDQ*, Bio9**)**, Min Temperature of Coldest Month (*MTCM*, Bio6**)**, Annual Mean Temperature (*AMT*, Bio1), Annual Precipitation (*AP*, Bio12), Precipitation of Wettest Quarter (*PWQ*, Bio16), Precipitation of Warmest Quarter (*PWQ*, Bio18), Temperature Seasonality (*TS*, Bio4), and Isothermality (*IS*, Bio3). The variables with highest loading values for PC2 and PC3 were *MTWaM* and temperature range (Bio2), respectively (Table 2). According to PC1, populations from north of the TM can be separated from that from south of TM (Fig. S3).

**Table 2:** Principle components analysis (PCA) of 19 climatic variables (bioclim) for the original locations of *Carex moorcroftii* populations.

	PC1	PC2	PC3
**Eigenvalue**	11.333	3.252	1.954
**Percentage variation explained Eigenvectors**	59.65%	17.12%	10.29%
**Eigenvectors**			
Annual Mean Temperature **(Bio1)**	0.897	0.437	−0.008
Mean Diurnal Range (Mean of monthly (max temp - min temp)) **(Bio2)**	0.002	−0.101	0.653
Isothermality (BIO2/BIO7) (* 100) **(Bio3)**	0.852	−0.252	−0.288
Temperature Seasonality (standard deviation *100) **(Bio4)**	−0.877	0.188	0.433
Max Temperature of Warmest Month **(Bio5)**	0.604	0.742	0.273
Min Temperature of Coldest Month **(Bio6)**	0.913	0.293	−0.271
Temperature Annual Range (BIO5-BIO6) **(Bio7)**	−0.788	0.173	0.585
Mean Temperature of Wettest Quarter **(Bio8)**	0.683	0.689	0.231
Mean Temperature of Driest Quarter **(Bio9)**	0.944	0.282	−0.129
Mean Temperature of Warmest Quarter **(Bio10)**	0.708	0.674	0.194
Mean Temperature of Coldest Quarter **(Bio11)**	0.950	0.262	−0.159
Annual Precipitation **(Bio12)**	0.895	−0.266	0.268
Precipitation of Wettest Month **(Bio13)**	0.848	−0.331	0.178
Precipitation of Driest Month **(Bio14)**	0.413	−0.482	0.530
Precipitation Seasonality (Coefficient of Variation) **(Bio15)**	−0.272	0.090	−0.263
Precipitation of Wettest Quarter **(Bio16)**	0.886	−0.266	0.273
Precipitation of Driest Quarter **(Bio17)**	0.782	−0.530	0.075
Precipitation of Warmest Quarter **(Bio18)**	0.881	−0.252	0.294
Precipitation of Coldest Quarter **(Bio19)**	0.720	−0.610	−0.123

### Genetic divergence

We found a total of 178 alleles across all loci and sampling plots. The mean number of effective alleles (*A*e) was 2.679 and ranged from 1.842 to 4.024. We noted that *C. moorcroftii* populations showed high genetic diversity, where *H*_O_ ranged from 0.480 to 0.806, with a mean value of 0.664 and *H*_e_ showed a mean value of 0.579, ranged between 0.409 and 0.732 (Table 3). Bayesian analyses of the full data (n = 638) revealed significant deviations from HWE and LD for multiple loci and populations. The probability of the presence of null alleles across loci was lower than 0.05% (<0.05%).

**Table 3:** Genetic diversity of 18 *Carex moorcroftii* populations along a latitudinal transect on the Qinghai-Tibetan Plateau.

Pop	*A*_e_	*H*_o_	*H*_e_	*F*	*N*_e_
P1	3.553(0.354)	0.625(0.047)	0.698(0.039)	0.081(0.050)	1173.6
P2	3.140(0.480)	0.673(0.074)	0.623(0.047)	−0.0762(0.103)	1075.4
P3	4.024(0.449)	0.732(0.040)	0.732(0.026)	−0.018(0.053)	1443.3
P4	2.149(0.166)	0.671(0.096)	0.521(0.030)	−0.269(0.156)	982.7
P5	3.325(0.298)	0.764(0.059)	0.678(0.036)	−0.149(0.073)	1219.7
P6	2.122(0.151)	0.626(0.085)	0.509(0.040)	−0.194(0.096)	983.1
P7	3.033(0.390)	0.623(0.054)	0.611(0.052)	−0.054(0.059)	1247.2
P8	2.624(0.241)	0.775(0.062)	0.594(0.033)	−0.334(0.106)	1397.9
P9	2.629(0.143)	0.758(0.076)	0.619(0.020)	−0.253(0.124)	1140.1
P10	2.562(0.283)	0.806(0.082)	0.573(0.042)	−0.422(0.137)	1170.4
P11	2.207(0.215)	0.711(0.102)	0.502(0.056)	−0.382(0.128)	898.6
P12	3.040(0.260)	0.800(0.061)	0.664(0.022)	−0.237(0.097)	1107.6
P13	1.842(0.180)	0.480(0.118)	0.409(0.052)	−0.032(0.179)	1289.9
P14	2.616(0.252)	0.653(0.088)	0.577(0.054)	−0.128(0.091)	1437.7
P15	2.085(0.179)	0.727(0.108)	0.473(0.065)	−0.542(0.114)	967.9
P16	2.250(0.160)	0.536(0.113)	0.536(0.041)	0.045(0.163)	1243.9
P17	2.848(0.374)	0.483(0.081)	0.592(0.058)	0.208(0.118)	1367.4
P18	2.169(0.152)	0.514(0.080)	0.518(0.043)	0.034(0.101)	1159.7
Mean	2.679(0.075)	0.664(0.020)	0.579(0.011)	−0.149(0.029)	

*A*_e_, number of effective alleles; *H*_o_, observed heterozygosity; *H*_e_, expected heterozygosity; *F*, fixation index. The F values with significant deviation from 0 are in bold.

AMOVA showed 72.3% of the genetic diversity existed within populations, 8.9% among populations, and 18.8% between regions (data not shown). Interestingly, *F*_st_ showed 27.8% of genetic diversity existed between regions, a larger estimate than AMOVA.

**Figure 1:**
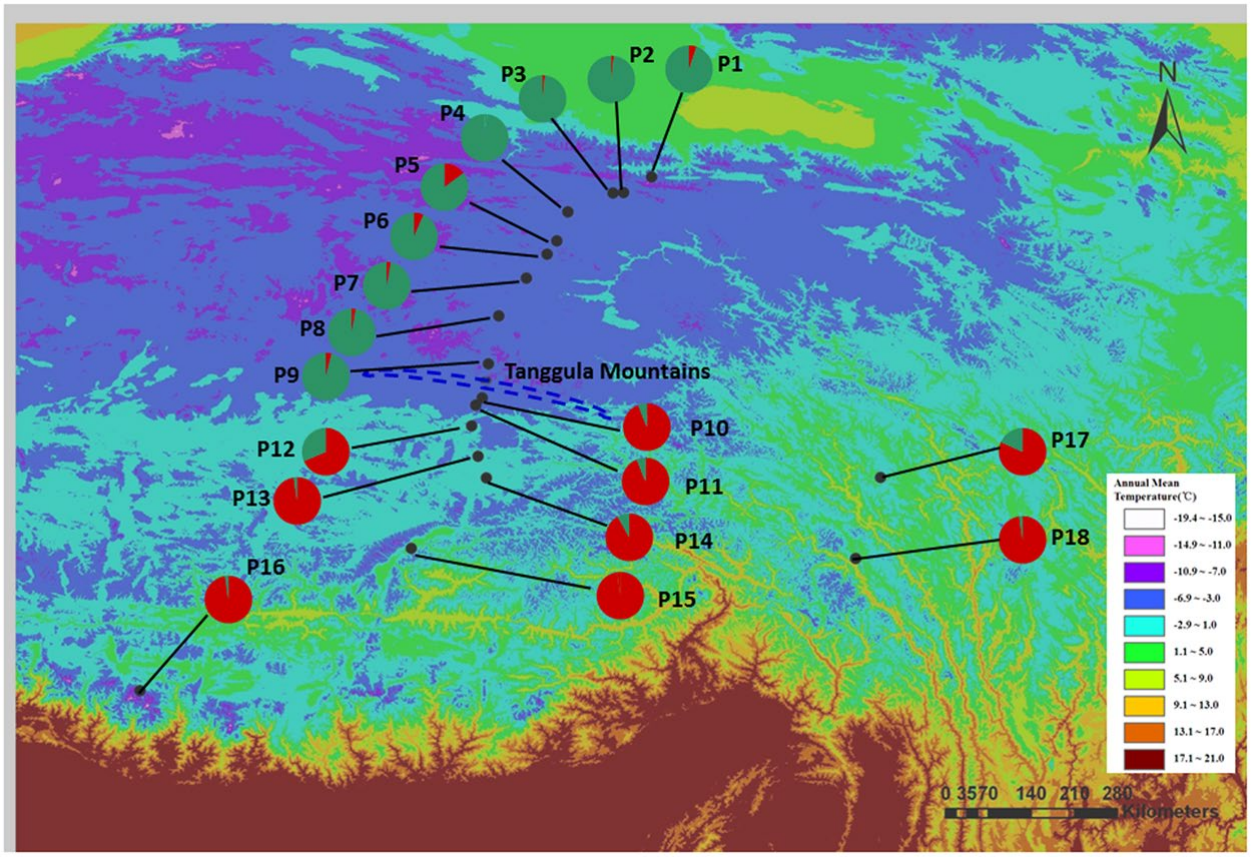
Sampling locations and genetic divergence of *Carex moorcroftii* populations illustrated by STRUCTURE. The pie charts indicates the genetic composition for each population based on the result of a representative STRUCTURE run with *K* = 2. Dashed line represent the Tanggula Mountains. Annual mean temperature data were downloaded from WorldClim Dataset (www.worldclim.org/bioclim). The map was processed by ArcGIS ver 10.2 (ESRI, Redlands, California, USA) (http://www.esri.com/).

**Figure 2:**
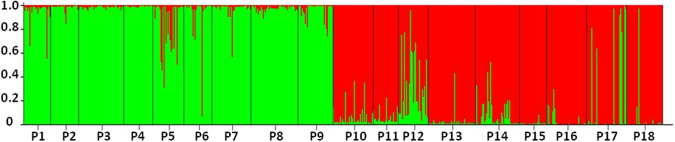
Population structure of *C. moorcroftii* of the northern (populations P1–P9) and southern mountainsides of Tanggula Mountains (populations P10–P18). Each population is represented by one chart, showing the relative proportion of membership to the different clusters for *K* = 2, as deduced from STRUCTURE analyses.

**Figure 3:**
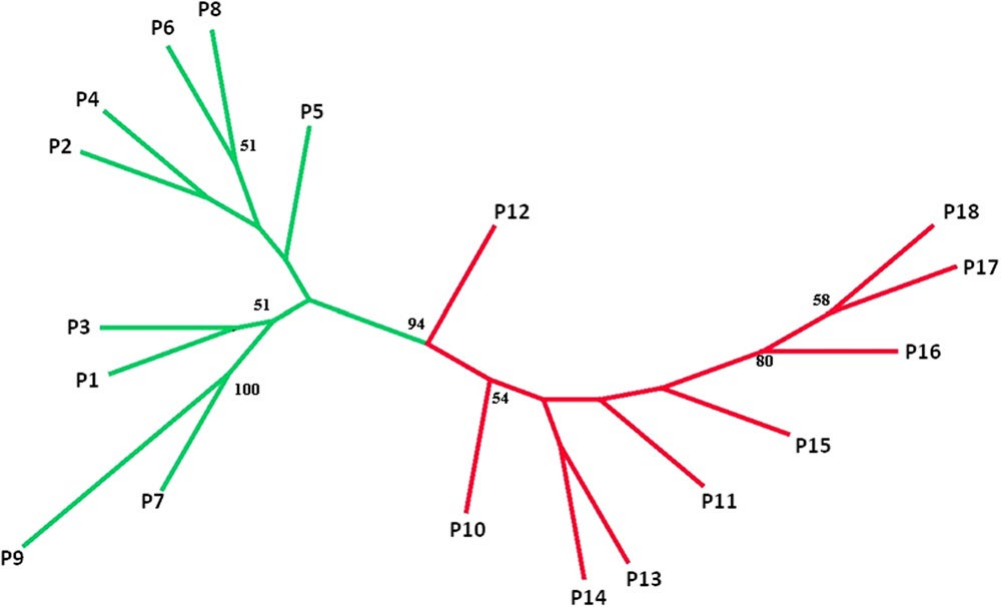
Unrooted neighbor-joining tree of *Carex moorcroftii* populations based on Nei’s genetic distance matrices. Numbers at nodes are significant bootstrap support percentages from 1,000 replicates on loci.

Our STRUCTURE analysis assigned the 18 populations into two genetic groups (Fig. S4): the populations from north of TM (P1-P9) grouped together (north group), and the others that from south of TM (P10-P18) formed the other group (south group) (Figs 1 and 2). The unrooted neighbor-joining tree showed a similar pattern as STRUCTURE (Fig. 3).

**Figure 4:**
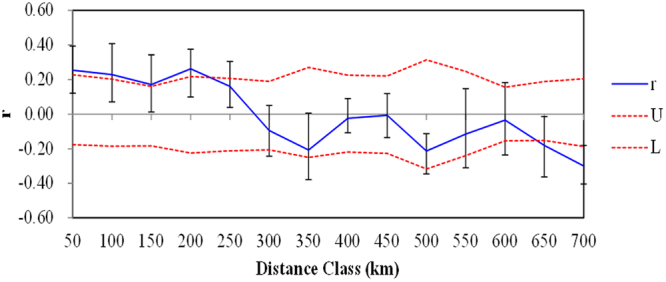
Correlograms showing the genetic correlation per distance class against geographical distance. Dotted lines indicate the 95% C.I. about the null hypothesis of random distribution of genotypes and individuals.

The autocorrelation analysis showed that there was significant spatial genetic structure (SGS) up to a distance class of 200 km (Fig. 4), which indicated that migratory effects could act as far as 200 km at a landscape scale.

### Gene flow

The mean value of pairwise *F*_st_ among populations from the north group was 0.143, and that of the south group was 0.269, both were lower than the value (0.278) between south and north groups (one-tailed Fisher’s Exact Test, p = 0.002) (Table S2). Accordingly, the gene flow within both groups was higher than that across the groups (1.50 (north group) and 0.68 (south group) *vs*. 0.65 (between groups)).

Coalescent-based Bayesian estimates by MIGRATE indicated that *C. moorcroftii* populations have relatively large effective population sizes with *Ne* > 890 (Table 3) and considerable inter-population historic gene flow (mean *Nm* = 1.119), which supports the estimate based on *F*_st_ values (*Nm* = 0.889) (Table S3). Estimates of recent migration between populations obtained with BAYESS ranged from 0.005 to 0.164, with a mean of 0.0099 (Table S4). In general, recent migration occurred mainly within population groups of the same side of TM, with 0.0170 of the migration rate occurring on the north group and 0.0094 on the south group, whereas 0.0069 occurred between inter- groups.

### Morphological variation

Among populations, 11 morphological traits differed significantly (Table 4). For example, the lowest variation range between populations was a 1.5-fold difference for *W*_leaf,_ ranging from 1.58 mm to 2.33 mm, whereas *M*_rhizomes_ showed a 15-fold difference and ranged from 0.81 g to 12.26 g (Table S5).

**Table 4:** Morphological characters, their abbreviation and measurement methods on *Carex moorcroftii* individuals.

Abbreviation	Full name	Measure methods
*L*_leaf_	Length of the leaf	Length from the tip to the bottom of the longest leaf (cm)
*W*_leaf_	Width of the leaf	Width of the widest part of the longest leaf (cm)
*N*_leaf_	Number of leaves per ramet	Number of leaves per ramet
*L*_rhizome_	Length of the rhizome	Length of the rhizome between neighbor internodes (cm)
*H*_infructescence_	Height of infructescence	Height from base to the tip of the infructescence (cm)
*M*_shoots_	Biomass of individual ramets	Dry weight of individual ramets (g)
*M*_rhizomes_	Biomass of individual rhizomes	Dry weight of rhizomes connected with the ramets (g)
*M*_roots_	Biomass of roots	Dry weight of roots connected with the rhizomes (g)
*M*_infructescence_	Biomass of infructescence	Dry weight of infructescence (g)
*M*_1000seeds_	Biomass per 1000 seeds	Dry weight of 1000 seeds (g)
*N*_seeds_	Number of seeds per infructescence	Number of seeds per infructescence
*N*_spikelet_	Number of spikelets per infructescence	Number of spikelets per infructescence
*M*_total_	Biomass of total plants	*M*_infructescence_ + *M*_shoots_ + *M*_rhizomes_ + *M*_roots_
*RAB*	Ratio of aboveground to belowground biomass	(*M*_infructescence_ + *M*_shoots_)/(*M*_rhizomes_ + *M*_roots_)
*RSV*	Ratio of sexual to vegetative biomass	*M*_infructescence_/*M*_shoots_

A PCA analysis on the 15 morphological traits showed that the first three principal components accounted for 71.3% of the total variance (Table 5). PC1, PC2, PC3 explained 34.6%, 20.9% and 15.8% of the total variance respectively. Biomass-related traits, e.g., *M*_rhizomes_, *M*_roots_, *M*_shoots_, *RSV*, *RAB*, and *M*_total_ had high loading values for PC1, reproductive traits, e.g., *M*_infructescence_, *M*_1000seeds_, *N*_seeds_, *N*_spikelet_ had high loading values for PC2, *N*_leaf_ and *H*_infructescence_ had high loading values for PC3. A scatterplot of PC1 against PC2 showed that the morphological variations of P2 and P8 are separated from other populations (Fig. S5).

**Table 5:** Principle components analysis (PCA) of 15 morphological traits for 11 *Carex moorcroftii* populations along the transect.

	PC1	PC2	PC3
Eigenvalue	5.184	3.128	2.376
Percentage variation explained	34.56%	20.85%	15.84%
Eigenvectors			
*L*_leaf_	0.482	0.346	−0.249
*W*_leaf_	−0.425	0.784	−0.170
*N*_leaf_	−0.049	0.254	−0.761
*L*_rhizome_	0.056	0.357	0.173
*H*_infructescence_	0.490	0.313	−0.712
*M*_rhizomes_	0.905	−0.174	0.223
*M*_roots_	0.837	0.403	−0.172
*M*_shoots_	0.872	−0.133	0.147
*M*_infructescence_	0.535	0.731	−0.349
*M*_1000seeds_	0.287	0.456	0.424
*N*_seeds_	−0.269	0.659	0.562
*N*_spikelet_	0.094	0.700	0.586
*RAB*	−0.750	0.456	0.022
*RSV*	0.622	−0.006	0.263
*M*_total_	0.920	−0.135	0.193

Abbreviations are the same as Table 4.

The Allometric models indicated that only population P1 and P5 possessed allometric allocation when we considered log*M*_shoots_ − log*M*_infructescence_ (Table S6). All 11 populations had significant positive combinations between log *M*_aboveground_ and log *M*_beloweground_, showing allometric allocation of biomass. The populations from north group (P2, P5, P7) invested more to aboveground organs during growth, with a slope significantly <1.0, whereas south group (P12, P15) displayed a reverse trend, with a slope >1.0 (Table S6).

Interestingly, most populations of *C. moorcroftii* (except P11, P12) showed some type of allometric allocation in log*M*_roots_ − log*M*_rhizomes_. The north populations had slopes <1.0 (P2, P7), and the south ones >1.0 (P10), indicating that the former invested more in roots than the later.

### Population variations associated with environmental factors

**Figure 5:**
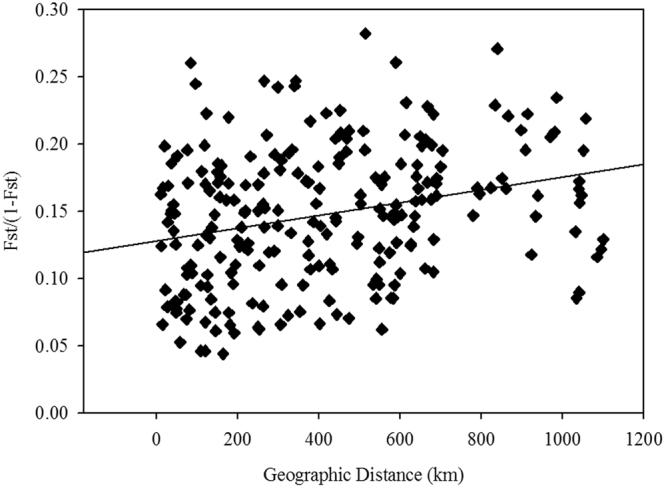
The relationship between genetic (FST/(1–FST)) and geographic distances between the analyzed populations of *C. moorcroftii*. (*R*^2^ = 0.107, *p* = 0.032)

Mantel-tests showed a weak but significant positive correlation between genetic distance (*GD*) and geographic distance (*GGD*) (*r* = 0.328, *p* = 0.032) (Fig. 5) and between genetic and environmental distance (*ED*) (*r* = 0.402, *p* = 0.005) (Table 6). When the influence of other factors were controlled, the genetic-geographical association was not significant (*r* = 0.072, *p* = 0.349) while the genetic-environmental association was also significant (*r* = 0.258, *p* = 0.045; Table 6). In addition, our MMRR analysis revealed that environmental isolation had a stronger influence on population divergence than distance isolation (β = 0.16 *vs*. 0.11, Table 6). The mantel and partial mantel tests further suggested that the environmental variables *AMT* (Bio1), *MTWaM* (Bio5), *MTCM* (Bio6), *MTDQ* (Bio9), *MTWaQ* (Bio10), *MTCQ* (Bio11) were main contributors to genetic divergence (Table S7, Fig. S6).

**Table 6:** Results of the Mantel test, partial Mantel test and MMRR analyzing of the correlation between geographical distances (*GGD*), environmental distances (*ED*), Nei’s genetic distance (*GD*) based on microsatellite data and differentiation in morphological traits (*MD*).

Matrix pair	Mantel test		Partial Mantel test			MMRR	
	*r*	*P*_value_	controlled	*r*	*P*_value_	β	*P*_value_
GD.GGD	0.328	0.032	*ED*	0.072	0.349	0.106	0.032
*GD*.*ED*	0.402	0.005	*GGD*	0.258	0.045	0.162	0.005
*MD.GGD*	0.137	0.184	*ED*	0.186	0.181	—	—
*MD.ED*	0.010	0.411	*GGD*	−0.127	0.349	—	—
*GD.MD*	0.358	0.039	—	—	—	—	—

The Mantel tests revealed no significant effects of *GGD* (*r* = 0.137, *p* = 0.184) or *ED* (r = 0.010, *p* = 0.411) on morphological differentiation (*MD*) (Table 6), whilst a significant effect of *GGD* on the differentiation of *N*_seeds_ (Table 7) was clear. Significant correlations between *MD* and *GD* were found in the traits *W*_leaf_, *N*_leaf_ and *RSV* (Table 7). However, when controlling for *GGD*, *W*_leaf_ and *N*_leaf_ still significantly correlated with *GD*.

**Table 7:** Mantel tests of the relationship between pairwise differentiation in morphological traits (*MD*) and partial Mantel tests controlling for pairwise differentiation in neutral genetic markers (*GD*) or geographic distance (*GGD*).

*MD*		*GGD*	*GD*	*GGD|GD*	*GD |GGD*
*W*_leaf_	r	0.073	**0.423**	−0.090	**0.425**
	p	0.274	**0.023**	0.336	**0.025**
*N*_leaf_	r	−0.132	**−0.263**	−0.043	−0.234
	p	0.247	**0.036**	0.424	0.091
*M*_roots_	r	0.233	0.216	0.171	0.147
	p	0.075	0.112	0.137	0.187
*N*_seeds_	r	**−0.250**	0.189	**−0.274**	0.222
	p	**0.026**	0.189	**0.017**	0.144
*RSV*	r	0.134	**0.435**	0.102	**0.428**
	p	0.165	**0.015**	0.218	**0.020**

Only the traits having significant relationships with *GGD* or/and *GD* were listed and the significant Mantel tests and partial Mantel tests were illustrated with bold fonts.

The partial Mantel tests between *MD* and three climatic principal components difference matrices (PCclim1 to PCclim3) controlling for GGD or *GD* revealed that *M*_roots_, *M*_infructescence_, *M*_1000seeds_, *N*_seeds_ and *RAB* were significantly influenced by climatic variables (PC1clim and PC2clim) (Table 8). Further mantel and partial mantel tests indicated that *M*_roots_ and *N*_spikelet_ were significantly influenced by *AMT* (Bio1), *H*_infructescence_ and *M*_roots_ by *MTWaM* (Bio5), *M*_roots_ and *M*_1000seeds_ by *MTDQ* (Bio9) and *MTCQ* (Bio11) (Table S7, Fig. S6).

**Table 8:** Mantel test between *MD* and principle components difference matrices of climate variables and partial Mantel tests for controlled pairwise *GD* and *GGD*, respectively. PCclim3 in Table 1 did not show correlation with any *MD* of traits.

*MD*	vs PCclim1		Controlling for *GD*		Controlling for *GGD*	
	*r*	*p*	*r*	*p*	*r*	*p*
***M***_**roots**_	**0.394**	**0.028**	**0.337**	**0.032**	**0.354**	**0.029**
*M*_infructescence_	0.155	0.210	0.088	0.309	0.219	0.188
*M*_1000seeds_	**0.371**	**0.046**	**0.384**	**0.048**	**0.454**	**0.030**
*N*_seeds_	**−0.278**	**0.047**	**−0.358**	**0.010**	−0.147	0.267
*RAB*	0.209	0.158	0.118	0.239	0.155	0.213
*MD*	**vs PCclim2**		*Controlling for GD*		*Controlling for GD*	
	*r*	*p*	*r*	*p*	*r*	*p*
*M*_roots_	**0.480**	**0.012**	**0.458**	**0.024**	**0.436**	**0.021**
*M*_infructescence_	**−0.247**	**0.047**	−0.212	0.094	−0.268	0.058
*M*_1000seeds_	−0.137	0.255	−0.136	0.287	−0.179	0.165
*N*_seeds_	−0.004	0.403	0.032	0.338	0.100	0.299
*RAB*	**0.559**	**0.025**	**0.542**	**0.005**	**0.549**	**0.011**

## Discussion

While population divergence is broadly driven by IBD or/and IBE, both theoretical and experimental studies suggest that in certain cases, genetic variation proportioning between populations reflects the consequence of the interactions of multiple factors^6,22^. Geographical barriers cause distributional gaps, leading not only to disjunct populations but also to cascading effects related to interrupted gene flow and habitat heterogeneity^2,21,23^. This study addressing these issues, showed that the high-altitude mountains of the TM acted as a geographical barrier to gene flow that enhanced population divergence in *C. moorcroftii*. Our results emphasize the importance of both gene flow and local adaptation in shaping population genetic structure.

### Genetic variation

Genetic variability reflects the potential capacity of plants to respond to environmental change^24^. We illustrated that *C. moorcroftii* held rich genetic variation (*H*_e = _0.58). The high level of genetic variability within populations of *C. moorcroftii* is similar to other outcrossing perennial grasses (e.g., *Stipa purpurea*, *H*_e_ = 0.585^25^) and comparable to those of the perennial and woody species that range across most areas of QTP (e.g., *Populus szechuanica*, *H*_e_ = 0.488^26^; *Prunus sibirica*, *H*_e_ = 0.774^27^). The high genetic diversity of *C. moorcroftii* may be due to its biological characteristics such as perenniality, wind-pollination, and generalist characteristics, allowing it to persist in a range of environmental conditions on the QTP. The relativly large effective population size (*N*e > 800, Table 3) and frequent gene flow between populations (mean *N*m > 1.0, Table S5) might also contribute to the observed high level of genetic diversity^25^. In addition, the *F* index data (Table 3) suggests that it has a high degree of outcrossing, hinting that sexual reproduction plays an important role in contributing to high genetic variability.

Significant heterozygote excess occurred in most *C. moorcroftii* populations (Table 3), suggesting a heterozygote advantage^25,28^. This may be attributed to faster growth and lower mortality of heterozygous genotypes compared to homozygous types, resulting in higher heterozygosity in populations^28^. The asexual propagation might also enable *C. moorcroftii* to contribute to the persistence of heterozygous genotypes in populations over the course of several sexual generations.

### Population genetic divergence and the effects of the TM on gene flow

Even though mantel tests (Fig. 5) and autocorrelation (Fig. 4) analysis indicated impacts from IBD, other analyses showed that the high altitude mountain TM played an important role on population divergence of the species. Specifically, all sampled *C. moorcroftii* populations can be divided into two groups according to the provenances from the south or north side of TM (Fig. 1). The similar pattern was also detected in our previous study by using ISSR markers^14^. Accordingly, the *F*_st_ values between populations on each side of the TM were lower than the global value (Table S2). Our estimation of gene flow further confirms the effect of the TM, with gene flow between populations on each side of the TM higher than across the TM (Table S4). For instance, the populations of P9 and P10 are close in geographic distance (only 27 km), and they are located on different sides of TM, the gene flow between them is lower than that between more distant populations on the same side. The pattern of gene flow strongly suggested that the TM may act as a geographical barrier weakening the genetic connection between populations on different sides (north or south) of the TM.

The reason for TM interrupted gene flow across north and south *C. moorcroftii* populations may be related to the snow–cover on the TM ridge and the dispersal modes of this species. The high elevation snow–covered ridge of the TM can directly impede air-mediated pollen flow in *C. moorcroftii* as a physical barrier, decreasing dispersal distance^25^. In addition, the seeds of *C. moorcroftii* have smooth surfaces, which mainly disperse by gravity to locations near mother plants. The pattern that physical barrier being a handicap to wind-mediated gene flow has also been found in other mountain species, such as *Ambystoma macrodactulym* and *Prunus armeniaca*, etc^29,30^. It is well established that gene flow has homogenizing effects on genetic variation between populations which can swamp the effects of local adaption. As gene flow declines, population divergence increases due to the effect of genetic drift or/and local adaptation under heterogeneous habitat conditions^31^. We detected a higher divergence level in *C. moorcroftii* between the two sides of TM than that across all populations, supporting the hypothesis that high-altitude mountains may be barriers to gene flow and then enhance population divergence.

Interestingly, TM’s effects on gene flow were not found in another dominant species of *Stipa purpurea* that is also crossing the TM and widespread on the QTP. Instead, a mosaic genetic structure existed in *S. purpurea*. Different seed dispersal modes may account for the different patterns of genetic divergence of these two species. Seeds of *S. purpurea* can be long-distance dispersed with the assistance of migratory herbivores, such as the chiru (*Pantholops hodgsonii*)^25^, whereas the seeds of *C. moorcroftii* were dispersed mainly by gravity, resulting in limited gene flow. The differences of population genetic structure between the two co-occurred sepcies additionally suggested the effectiveness of our sample strategy of avoiding the effects of human transport on genetic estimation for the plant species nearby a highway.

### Population morphological divergence

The PCA of 15 morphological traits for *C. moorcroftii* populations revealed the existence of morphological variations between populations. Further analyses found significant allometric allocations between several morphological traits, e.g. log*M*_shoots_ and log*M*_infructescence_, and log*M*_roots_ and log*M*_rhizomes_ (Table S6), suggesting trade-offs in plant structure growth among populations. Of particular interest is the allometric relationship between log*M*_aboveground_ and log*M*_belowground_, which indicated that populations invested resources in above- and below- ground biomass based on their location on the TM. Specifically the north populations allocated more resource to belowground structures, whereas the south ones favoured aboveground biomass investment. It is likely that this pattern reflects the difference in soil water availability since the north side has less precipitation than the south (Table S1). A similar pattern was also found in populations of *Carex bigelowii* at a single site that differed in soil humidity, where the wettest population had longest rhizomes^32^. Thus, we suppose the topography of TM has partially led to unique environmental conditions on each side and has directly impacted the morphological performance of populations (Fig. S5). Mantel tests further support this conjecture, which detected significant correlation between morphological variations (that of *M*_roots_, *M*_infructescence_, *M*_seeds_, *N*_seeds_ and *RAB*) and climatic variables (PC1clim & PC2clim) (Table 8). Other studies have also provided evidence of this pattern, for example Zhong *et al*.^33^ detected decreasing leaf width of *C. moorcroftii* with altitude and Stenstrӧm *et al*.^34^ found that morphological traits of four sedges (*Carex bigelowii*, *C. ensifolia* subsp. *arctisibirica*, *C. lugens*, *C. stans*) changed with latitude. These findings together suggest the morphological response of plant populations to environmental change. Further common garden trials may provide more direct evidence of morphological adaption or/and phenotypic plasticity in response to environmental variation.

### Signal of IBE

After establishing evidence of population divergence due to the effects of the TM and IBD, we showed that the genetic distance between *C. moorcroftii* populations was positively correlated with PCclim1 both when GGD was controlled or not (Table 6), suggesting the influence of IBE. Due to the difficulties in high altitude environments, we could not conduct common garden trials to determine whether local adaptation to site-specific habitat exists in the surveyed populations. The overall environmental variables (variation averaged across all environmental variables after standardization) were obvious (Table 6), and we found that morphological variations correlated with climate variables (Tables 7, 8).

Thus, we suggested that the contribution of climatic variables to habitat heterogeneity may lead to IBE. A similar assumption has been suggested in other studies, particularly Wang & Bradburd^8^, Wu *et al*.^35^, and Sexton *et al*.^36^. A driver of IBE patterns may be attributed to a neutral process of temporally disrupted gene flow among individuals living in environmentally distinct habitats^2^. Environmentally distinct habitat in turn can act as a barrier to gene flow, causing environmental isolation and genetic differentiation between spatially close populations^6^. As the highest place in the world, QTP exhibits a large amount of heterogeneity in environmental conditions^14^, such as precipitation, temperature, snow-cover duration, and topography, which differ among *C. moorcroftii* populations on QTP. These environmental differences impact morphological traits expressed by plants, such as phenology^6,35^. We recommend further study of other plant species on the QTP to provide more insights into the mechanisms of IBE.

### Implications

Global climate change is now one of the most important problems currently challenging the world. With the influence of climate on genetic variation and population performance, the impact of climate change is especially concerning for plants on QTP. Our findings suggest that, although the high-altitude mountains of TM acted as a barrier to gene flow and enhanced population divergence of *C. moorcroftii* between the north and south faces of TM, this species holds rich genetic diversity, large effective population sizes and considerable inter-population gene flow. These features along with outcrossing may counteract the effects of genetic drift, thus maintaining and transferring adaptive variation among populations in response to continuous climate change^25,37^.

In addition, morphological and genetic divergence among populations is associated with climatic factors, especially adaptive strategy in response to soil moisture divergence between north and south sides of TM by a trade-off between below and above growth, suggesting adaptive potential of *C. moorcroftii*. Such information is highly useful in modelling future vegetation dynamics under climate change.

## Methods

### Study species

*Carex moorcroftii* Falc. ex Boott (Cyperaceae) is a member of the section Racemosae G. Don in the subgenus *Carex* Linnaeus^38^. It grows in alpine areas of Central Asia and Tibet, with the core of its distribution on the QTP. The altitudinal range of this plant covers 3,400–5,700 m^16,39^ and is one of the dominant species of alpine grasslands and meadows on the QTP. *C. moorcroftii* is a perennial sedge and reproduces both asexually, by elongating rhizomes and forming clonal ramets, and sexually, where it flowers and produces seeds from July to September^16^.

### Ecological niche modeling (ENM)

The range of *C. moorcroftii* was estimated based on current climatic layers used in the maximum-entropy algorithm (MaxEnt 3.3)^40^. MaxEnt is a common modeling algorithm used for presence-only data, which calculates the probability of presence, based on occurrence locations relative to random background conditions called pseudo absences. We used occurrence data from three sources; 86 records from the Chinese Virtual Herbarium (CHV) (http://www.cvh.org), 188 from the Global Biodiversity Information Facility (GBIF) (http://www.gbif.org), and 25 from the published studies (e.g., Liu *et al*.^14^) or the authors’ collections for a total of 299 sampling points. We used records from CHV and GBIF with the taxonomic annotation “*Carex moorcroftii*” with the exception of records with unclear location descriptions. The environmental layers used were the 19 bioclimatic variables (1950–2000) obtained from Worldclim (http://www.worldclim.org/), with a resolution of 2.5 arc-minutes (5 km). We defined the extent of QTP according to the commonly accepted range of the QTP, between 73.31°E to104.78°E, 26.00°N to 39.78°N^41^.

To avoid potential over-parameterization of the model, we based the summary model predictions on 20 replications using a subsample method. For each replicate a 75% random selection of the occurrence locations were used for model training and the remaining 25% for model testing, with 20 replicates for each. We also set a regularization multiplier of 10, 10, 000 as background points, 5,000 numbers of maximum iterations and 0.00001 of convergence threshold. We quantified overfitting by comparing threshold-dependent omission rates with theoretically anticipated levels of omission. We used AUC (the area under ROC (the receiver operator characteristic curve)) to evaluate model performance^40^, where a perfect model has an AUC of 1, although a good model has an AUC > 0.7. To test the relative importance of each climatic variable, we applied both percentage contribution and permutation approaches. Maxent creates marginal response curves and single variable response curves for each bioclimatic variables. Additionally, jackknife tests were used to determine the relative importance and co-linearity among variables using the training gain, test gain, and test AUC.

### Sampling design

ENM revealed that Max Temperature of Warmest Month (*MTWaM*, Bio5), Mean Temperature of Warmest Quarter (*MTWaQ*, Bio10) and Temperature Seasonality (standard deviation*100) (*TS*, Bio4) were the most important factors associated with the distribution of *C. moorcroftii* (date not shown). A north-south transect crossing the TM on the QTP was designed to sample *C. moorcroftii* populations. Along this transect, we sampled a total of 18 populations, ranging from N 28° to 35° in latitude, E 86° to 97° in longitude and 4,100–5,100 m in altitude (Fig. 1; Table 1). These populations spanned across 2,000 km and several climatic gradients: a temperature gradient characterized by a 7.7 °C range in *MTWaM* (11.0–17.7) and 6.2 °C difference in *MTWaQ* (4.9–11.1 °C) a seasonality gradient, *TS* varied from 6.25 to 9.03 °C across the sample sites by using the raster data (Table S1). To reduce the influence of highway as possible, all sample plots we chose were away from the highway at least 2.0 km (2~5 km) and where the vegetation was intact.

We collected plant materials of *C. moorcroftii* in August 2015 when the plants were fruit- and seed-setting. For population genetic analyses, approximately 40 individuals per population were randomly sampled except in cases where there weren’t enough individuals (Table 1). To avoid collecting from the same clone, the minimum distance between sampled individuals was 4 m. For each sample, fresh leaves were collected, and desiccated using silica gel and kept at −20 °C until DNA extraction.

As a clonal plant, *C. moorcroftii* possesses rhizomal guerilla growth, making it difficult to identify the entire individual plant belonging to the same clone (genet). To measure morphological traits, we sampled using the quadrat method following Sun *et al*.^42^. For each population, we first set up a transect 100 m long with one quadrat plot of 1.0 m × 1.0 m for every 10 m along this transect. After evaluating the number of ramets and flowering shoots for each plot, we collected all underground plant material for *C. moorcroftii* in each plot to a depth of 1.0 m. Thus, each population included 10 samples for phenotypic trait variation analysis. Of the 18 DNA-sampled populations, seven (Suonandajie (P3), Wudaoliang (P4), Tuotuohe station (P6), Naqu (P13), Everest 4900 (P16), Yela mountain (P17), Chuanzangxian (P18)) did not have adequate fruiting samples (<3). To avoid the statistical bias associated with small sample sizes, the seven populations were not included in the analysis of morphological variations (Table S5).

### Phenotypic trait measurement

Plant material for each plot was separated and classified as: leaves, roots, spikes, or rhizomes. We measured traits related to performance: length of leaf (*L*_leaf_), rhizome (*L*_rhizome_) and infructescence (*H*_infructescence_); weight of leaf (*W*_leaf_); and the number of leaves (*N*_leaf_), Spikelet (*N*_spikelet_) and seeds (*N*_seeds_). We dried the plant material in an oven at 60 °C for 48 h and then weighed it. All measured parameters and methods are listed in Table 4.

### SSR assay

DNA was extracted from 25–30 mg of dried leaf material following the methods from Doyle & Doyle^43^ with a minor modification. Using Liu *et al*.^44^ as a reference, we selected 12 pairs of SSR primers, fluorescently labeled the forward primers with Fam, Rox, and Hex, which were produced by Sangon Biotech (Shanghai, China). PCR was performed in 10 μl volume, which included ca. 20 ng of DNA, 0.2 μM of primers, 2.5 mM of MgCl_2_, 0.2 mM of dNTP (Sangon Biotech (Shanghai, China)), 1 × Taq of buffer, and 0.5 U of Taq polymerase. The PCR was carried out in a 2720 Thermal Cycler (Applied Biosystems, Foster City, CA, USA: ABI) and proceeded as follows: 4 min at 94 °C; then 35 cycles for 45 s at 94 °C, 30 s at 55–62 °C, 45 s at 72 °C; then a final extension step of 10 min at 72 °C. Alleles were sized on a Hitachi ABI 3730 (ABI) automated sequencer using LIZ 500 (ABI) as a ladder and analysed in Genemapper 4.0 (ABI).

### Data Analysis

Although the ENM forecasted that *MTWaM* and *MTWaQ* were the two most important environmental variables for *C. moorcroftii*, the habitat heterogeneity at a regional scale can also be associated with the other 17 bioclimatic variables. The top contributing variables are the variables where the average at the occurrence locations is the most different from the background. Thus, these two variables alone do not adequately describe variation in the climatic aspect of the habitat. However, that being said the least contributing variables have averages at occurrence locations that are not different from the background, so they probably have a similar range of values and therefore may not be a good choice to explain variation in the climatic conditions of the species’ distribution. Thus, we used principal component analysis (PCA) to reduce the dimensionality of all 19 of the bioclimatic variables for the original locations of *C. moorcroftii* populations to infer habitat heterogeneity.

### Morphological trait

Descriptive statistical analyses were performed to characterize the morphological variations of the 11 trait-measured populations (Table 4). A principal component analysis (PCA) was performed to reduce the dataset to set interrelated traits using SPSS v19.0.

We determined the proportion of reproductive biomass (R) to vegetative biomass (V) and also shoot biomass (A) to root biomass (U) by using the classical allometric model, R = aVb, fitted as log R = log a + b log V and A = aUb, and fitted as log A = log a + b log U^45^, where parameter a is referred to as the ‘allometric coefficient’ and b the ‘allometric exponent’. An exponent significantly different from 1.0 indicates an allometric (non-isometric/proportional) relationship. We used standardized major axis (SMA) regression to fit the allometric relationship and estimate the parameters, and tested whether the slope of each population statistically differed from 1.0. We used the software package Standardised Major Axis Tests and Routines (SMATR)^46^ to conduct the SMA analyses. A *p* < 0.05 was used to determine statistical significance for heterogeneity slopes and slope differences.

### Genetic diversity

We used 1,000 randomizations in the program MICROCHECKER v2.2.3^47^ to detect the presence of null alleles and genotyping errors such as large allele dropout or stuttering. The effective number of alleles (*A*_e_), observed heterozygosity (*H*_O_), and expected heterozygosity (*H*_e_) were calculated by using GenAlex 6.41 software^48^. Hardy-Weinberg Equilibrium (HWE) and linkage disequilibrium tests (LD) were conducted for each population, and p-values were subjected to the sequential Bonferroni test for multiple comparisons by using GENEPOP v4.0^49^.

### Genetic structure

We analyzed the genetic structure of the *C. moorcroftii* populations in three distinct analyses. First, we estimated the genetic differentiation of *C. moorcroftii* populations using STRUCTURE 2.3^50^ to explore potential substructure by evaluating the number of subpopulations (*K*). Population numbers from 1 to 18 and each was tested 20 times at the population level based on 25,000-step burn-in and 100,000 MCMC iterations. We estimated the most probable number of gene pools (*K*) in the data by comparing the log probability of the data [Pr(X/K)] for each value of *K* across all 18 runs of STRUCTURE. The most probable number of subpopulations (*K*) was calculated using the Δ*K* statistic, which calculates the log-probability rate of change between successive analyses of *K*^51^. Secondly, we constructed Nei’s genetic distance matrices after creating 1,000 bootstrapped matrices using the program, Microsatellite Analyser (MSA)^52^. Thirdly, overall and pairwise estimates of differentiation (*F*_st_), were calculated by FSTAT^53^ and the significance of AMOVA output was tested with 999 permutations by using GenAlex 6.41software^48^.

### Gene flow

To compare the level of gene flow between both sides of the TM, we estimated pairwise *F*-statistics between all combinations of north - south population pairs.

To quantify average recent migration rates (last few generations) among populations, we applied a Bayesian approach using the software BAYESASS 1.3^54^. This method makes no assumptions of HWE within the sample and is based on transient multi-locus disequilibrium in multi-locus disequilibrium in multi-locus genotypes of migrants relative to the host population. It calculates inbreeding coefficients for each population separately and the joint probabilities are used to estimate recent migration rates. For our analysis we used the following parameters: a run length of 3,000,000 iterations with a 1,000,000 burn-in. a sampling frequency of 2,000, δp (maximum change of allele frequency between iterations) at 0.30, a δm of 0.15 (maximum change of migration rate) and a δF of 0.15 (maximum change of inbreeding coefficient). The dataset was run 10 times with different starting seeds to ensure consistency of results and the average of these runs is reported.

To estimate the direction and magnitude of historical gene flow between populations, the maximum likelihood (ML) approach implemented in MIGRATE version 3.2.7^55^ was applied. The ML model assumes that populations are at drift-migration equilibrium, have a constant size and migration occurs over the coalescent period (approx. 4Ne generations). However, unlike *F*-statistics, this method allows non-symmetrical migration and differences in size among populations, both of which biologically plausible scenarios. MIGRATE jointly estimates the mutation-scaled effective population sizes (θ = 4Neµ, where µ is the mutation rate for diploid data) and the mutation-scaled effective immigration rate (M = m/µ, where m is the immigration rate) between geographical groups. MIGRATE accomplishes this by estimating allele genealogies and approximating the sum of probabilities across possible genealogies using the Metropolis-Hastins Markov chain Monte Carlo sampling. The Brownian motion model for microsatellite as an approximation of the ladder model was used as the mutation model. As a search strategy, ten short chains with 10,000 recorded trees, followed by three long chains with 50,000 recorded trees with a burn-in of 20,000 and a sampling increment of 100 was used. The first run estimated θ and M from *F*_st_ values, whereas subsequent runs incorporated the ML estimates of θ and M from the previous run as the starting parameter values converged. We used the results of the fifth run for interpretation. To avoid problems due to different sample sizes among groups, a reduced random subset (37 individuals corresponding to the lowest group size) of each population was used in a parallel computation.

In addition, we used GenAlex v6.41 to estimate spatial autocorrelation at the landscape scale distribution of gene flow in *C. moorcroftii* populations. The distance class was set to 50 km, and the autocorrelations were performed using 1,000 permutations.

### Correlation between population divergence estimated based on morphological and genetic variations

To test for correlations between morphological (*MD*), genetic (*GD*), geographical (*GGD*), and environmental (*ED*) distances among populations, Mantel tests were performed using the Spearman rank correlation coefficient. We calculated Euclidean distances on standardized values ($[{\rm{x}}-\bar{{\rm{x}}}]/{\rm{SD}}$) for all 15 morphological variables to determine MD. We used the pairwise *F*_st_ as GD. We reduced the 19 bioclimatic environmental variables to components of principal component analysis (PCA) and the Euclidean distance calculated with the values of the PCA axes was considered the ED. To perform the Mantel test between *MD* and *GD*, we only used populations shared by each of these distances, which excluded populations P3, P4, P6, P13, P16, P17, P18. If both *GGD* and *GD* were significantly correlated with *MD*, then partial Mantel tests were performed to evaluate their relative importance to morphological traits using the three climatic principal components difference matrices (PC1clim to PC3clim) after controlling for *GGD* or *GD*.

In addition, to test for possible spatial effects of genetic structure, isolation by distance (IBD) and isolation by environmental distance (IBE) were estimated. The correlations between genetic differentiation and geographic/environmental factors were determined by a combination of partial mantel tests and matrix regression analysis on distance matrices. Specifically, partial mantel tests between populations were performed between two factors of interest given the other variables in the model, as implemented in *R* using the “ecodist” package. Multiple matrix regression with randomization (MMRR) is a novel and robust approach for estimating the independent effects of potential factors, especially in situations of low-to-moderate gene flow. We implemented MMRR with 10,000 permutations in R with making use of the MMRR function script.

## Electronic supplementary material

Dataset 1
