# Supplementary material for: The Tanggula Mountains enhance population divergence in Carex moorcroftii: a dominant sedge on the Qinghai-Tibetan Plateau
Source: Sci Rep. 2018 Feb 9;8:2741. doi: 10.1038/s41598-018-21129-y (PMC5807306; doi:10.1038/s41598-018-21129-y)
Supplement: Supplementary file 1 — Dataset 1 [file 41598_2018_21129_MOESM1_ESM.doc]

**The Tanggula Mountains enhance population divergence in *Carex moorcroftii*: a dominant sedge on the Qinghai-Tibetan Plateau**

Wensheng Liu^1, 2, 3^, Yao Zhao^1^, Danhui Qi^3^, Jianling You^1^, Yin Zhou^1^, Zhiping Song^1※^

**Supplementary Table S1 19 climatic variables of 18 *Carex moorcroftii* populations on the Qinghai-Tibetan Plateau.**

| **Code** | **Bio1** | **Bio2** | **Bio3** | **Bio4** | **Bio5** | **Bio6** | **Bio7** | **Bio8** | **Bio9** | **Bio10** | **Bio11** | **Bio12** | **Bio13** | **Bio14** | **Bio15** | **Bio16** | **Bio17** | **Bio18** | **Bio19** |
| --- | --- | --- | --- | --- | --- | --- | --- | --- | --- | --- | --- | --- | --- | --- | --- | --- | --- | --- | --- |
| P1 | -2.80 | 14.29 | 37.03 | 903.18 | 15.30 | -23.30 | 38.60 | 8.02 | -13.12 | 8.02 | -14.05 | 164 | 42 | 1 | 112.16 | 108 | 3 | 108 | 3 |
| P2 | -5.03 | 13.99 | 37.51 | 864.11 | 12.50 | -24.80 | 37.30 | 5.37 | -14.70 | 5.37 | -15.70 | 222 | 58 | 1 | 114.61 | 148 | 3 | 148 | 4 |
| P3 | -4.74 | 14.01 | 37.76 | 860.77 | 12.70 | -24.40 | 37.10 | 5.62 | -14.35 | 5.62 | -15.37 | 220 | 58 | 1 | 115.58 | 148 | 3 | 148 | 4 |
| P4 | -4.93 | 13.88 | 38.56 | 830.21 | 12.10 | -23.90 | 36.00 | 5.07 | -14.03 | 5.07 | -15.13 | 266 | 72 | 1 | 118.75 | 181 | 3 | 181 | 4 |
| P5 | -4.56 | 14.36 | 39.23 | 843.24 | 12.60 | -24.00 | 36.60 | 5.57 | -13.88 | 5.57 | -14.93 | 279 | 78 | 1 | 120.17 | 192 | 3 | 192 | 4 |
| P6 | -5.09 | 14.63 | 39.63 | 846.98 | 12.10 | -24.80 | 36.90 | 5.10 | -14.45 | 5.10 | -15.45 | 284 | 82 | 1 | 122.13 | 198 | 3 | 198 | 4 |
| P7 | -3.68 | 15.34 | 40.37 | 869.47 | 13.90 | -24.10 | 38.00 | 6.73 | -13.42 | 6.73 | -14.35 | 281 | 85 | 0 | 125.54 | 200 | 2 | 200 | 4 |
| P8 | -4.45 | 15.08 | 40.09 | 857.28 | 12.90 | -24.70 | 37.60 | 5.83 | -14.00 | 5.83 | -14.97 | 305 | 89 | 1 | 123.17 | 215 | 5 | 215 | 6 |
| P9 | -5.04 | 14.79 | 39.87 | 835.44 | 11.80 | -25.30 | 37.10 | 4.97 | -14.32 | 4.97 | -15.33 | 343 | 94 | 1 | 120.33 | 237 | 5 | 237 | 6 |
| P10 | -4.46 | 14.83 | 39.85 | 833.89 | 12.40 | -24.80 | 37.20 | 5.50 | -13.75 | 5.50 | -14.77 | 353 | 95 | 2 | 119.08 | 242 | 6 | 242 | 6 |
| P11 | -3.67 | 14.88 | 39.90 | 832.34 | 13.20 | -24.10 | 37.30 | 6.25 | -12.97 | 6.25 | -14.00 | 355 | 95 | 2 | 119.54 | 244 | 6 | 244 | 6 |
| P12 | -2.81 | 14.93 | 40.12 | 828.92 | 13.90 | -23.30 | 37.20 | 7.03 | -12.12 | 7.03 | -13.13 | 366 | 97 | 2 | 118.65 | 250 | 6 | 250 | 6 |
| P13 | -1.62 | 15.18 | 40.71 | 818.27 | 14.90 | -22.40 | 37.30 | 8.03 | -10.10 | 8.03 | -11.88 | 387 | 101 | 2 | 118.37 | 264 | 7 | 264 | 7 |
| P14 | -0.73 | 15.33 | 41.20 | 807.02 | 15.60 | -21.60 | 37.20 | 8.78 | -9.08 | 8.78 | -10.90 | 400 | 103 | 2 | 117.61 | 272 | 7 | 272 | 7 |
| P15 | 2.51 | 14.49 | 42.50 | 728.70 | 17.70 | -16.40 | 34.10 | 11.12 | -5.02 | 11.12 | -6.63 | 330 | 99 | 0 | 136.11 | 248 | 2 | 248 | 2 |
| P16 | -2.82 | 14.66 | 46.53 | 632.90 | 11.00 | -20.50 | 31.50 | 4.35 | -9.38 | 4.88 | -10.52 | 311 | 104 | 1 | 136.30 | 232 | 9 | 224 | 12 |
| P17 | 1.75 | 13.56 | 44.45 | 625.39 | 15.40 | -15.10 | 30.50 | 9.15 | -5.15 | 9.15 | -6.08 | 539 | 120 | 2 | 98.76 | 323 | 11 | 323 | 12 |
| P18 | 1.10 | 14.68 | 44.47 | 671.94 | 15.90 | -17.10 | 33.00 | 9.02 | -6.52 | 9.02 | -7.38 | 512 | 114 | 2 | 103.07 | 312 | 9 | 312 | 10 |

Notes: Bio1, Annual Mean Temperature; Bio 2, Mean Diurnal Range (Mean of monthly (max temp - min temp)); Bio 3, Isothermality (BIO2/BIO7) (* 100); Bio 4, Temperature Seasonality (standard deviation *100); Bio 5, Max Temperature of Warmest Month; Bio 6, Min Temperature of Coldest Month; Bio 7, Temperature Annual Range (BIO5-BIO6); Bio 8, Mean Temperature of Wettest Quarter; Bio 9, Mean Temperature of Driest Quarter; Bio 10, Mean Temperature of Warmest Quarter; Bio 11, Mean Temperature of Coldest Quarter; Bio 12, Annual Precipitation; Bio 13, Precipitation of Wettest Month; Bio 14, Precipitation of Driest Month; Bio 15, Precipitation Seasonality (Coefficient of Variation); Bio 16, Precipitation of Wettest Quarter; Bio 17, Precipitation of Driest Quarter; Bio 18, Precipitation of Warmest Quarter; Bio 19, Precipitation of Coldest Quarter.

**Supplementary Table S2 Pairwise *F*_st_ comparisons between all sampling sites above the diagonal as calculated by FSTAT version. (Goudet 1995).**

| **Pop** | **P1** | **P2** | **P3** | **P4** | **P5** | **P6** | **P7** | **P8** | **P9** | **P10** | **P11** | **P12** | **P13** | **P14** | **P15** | **P16** | **P17** | **P18** |
| --- | --- | --- | --- | --- | --- | --- | --- | --- | --- | --- | --- | --- | --- | --- | --- | --- | --- | --- |
| **P1** | - |  |  |  |  |  |  |  |  |  |  |  |  |  |  |  |  |  |
| **P2** | 0.1432 | - |  |  |  |  |  |  |  |  |  |  |  |  |  |  |  |  |
| **P3** | 0.094 | 0.1088 | - |  |  |  |  |  |  |  |  |  |  |  |  |  |  |  |
| **P4** | 0.1894 | 0.1407 | 0.1533 | - |  |  |  |  |  |  |  |  |  |  |  |  |  |  |
| **P5** | 0.0767 | 0.0729 | 0.0779 | 0.1481 | - |  |  |  |  |  |  |  |  |  |  |  |  |  |
| **P6** | 0.1722 | 0.1283 | 0.1438 | 0.2009 | 0.1362 | - |  |  |  |  |  |  |  |  |  |  |  |  |
| **P7** | 0.1421 | 0.1019 | 0.1139 | 0.2317 | 0.1238 | 0.1341 | - |  |  |  |  |  |  |  |  |  |  |  |
| **P8** | 0.1198 | 0.1365 | 0.1161 | 0.2033 | 0.1089 | 0.1206 | 0.1557 | - |  |  |  |  |  |  |  |  |  |  |
| **P9** | 0.1267 | 0.1653 | 0.138 | 0.2685 | 0.1193 | 0.2166 | 0.1306 | 0.1759 | - |  |  |  |  |  |  |  |  |  |
| **P10** | 0.2131 | 0.2518 | 0.1984 | 0.3319 | 0.2213 | 0.2958 | 0.2292 | 0.2524 | 0.2638 | - |  |  |  |  |  |  |  |  |
| **P11** | 0.2512 | 0.2864 | 0.245 | 0.3921 | 0.2046 | 0.384 | 0.2754 | 0.2973 | 0.2603 | 0.2892 | - |  |  |  |  |  |  |  |
| **P12** | 0.1312 | 0.1825 | 0.1456 | 0.2273 | 0.1298 | 0.2348 | 0.1907 | 0.1883 | 0.1892 | 0.1493 | 0.2537 | - |  |  |  |  |  |  |
| **P13** | 0.3613 | 0.3654 | 0.3216 | 0.3923 | 0.2982 | 0.4133 | 0.3862 | 0.3271 | 0.383 | 0.3985 | 0.4351 | 0.3277 | - |  |  |  |  |  |
| **P14** | 0.1683 | 0.2665 | 0.221 | 0.3055 | 0.1887 | 0.2819 | 0.2301 | 0.239 | 0.2149 | 0.2307 | 0.2931 | 0.1789 | 0.2886 | - |  |  |  |  |
| **P15** | 0.2935 | 0.3274 | 0.2787 | 0.4094 | 0.2776 | 0.4177 | 0.3118 | 0.2886 | 0.3202 | 0.2886 | 0.2543 | 0.2437 | 0.3889 | 0.2836 | - |  |  |  |
| **P16** | 0.2371 | 0.323 | 0.2653 | 0.3548 | 0.257 | 0.3413 | 0.2981 | 0.2951 | 0.3221 | 0.3389 | 0.3366 | 0.2573 | 0.3739 | 0.2002 | 0.3241 | - |  |  |
| **P17** | 0.2114 | 0.2793 | 0.2274 | 0.3095 | 0.1983 | 0.3271 | 0.2852 | 0.2465 | 0.2793 | 0.2607 | 0.2604 | 0.1593 | 0.3227 | 0.1833 | 0.232 | 0.1611 | - |  |
| **P18** | 0.2418 | 0.3395 | 0.2628 | 0.358 | 0.2424 | 0.3575 | 0.3201 | 0.2729 | 0.314 | 0.2968 | 0.345 | 0.2 | 0.3618 | 0.1857 | 0.281 | 0.1643 | 0.1206 | - |

**Supplementary Table S3 Effective population size of *Carex moorcroftii* in 18 studied populations estimated by MIGRATE.**

| **Pop** | **θ** | **P1** | **P2** | **P3** | **P4** | **P5** | **P6** | **P7** | **P8** | **P9** | **P10** | **P11** | **P12** | **P13** | **P14** | **P15** | **P16** | **P17** | **P18** |
| --- | --- | --- | --- | --- | --- | --- | --- | --- | --- | --- | --- | --- | --- | --- | --- | --- | --- | --- | --- |
| **P1** | 1.1736 | - | 1.100 | 1.338 | 1.098 | 0.988 | 0.997 | 1.005 | 1.35 | 1.133 | 1.534 | 0.982 | 1.017 | 1.061 | 1.118 | 1.32 | 1.26 | 1.189 | 1.181 |
| **P2** | 1.0754 | 0.949 | - | 1.395 | 0.871 | 0.835 | 1.159 | 1.445 | 1.272 | 1.289 | 1.642 | 1.211 | 1.271 | 1.014 | 1.34 | 0.83 | 1.083 | 1.234 | 1.102 |
| **P3** | 1.4433 | 0.878 | 0.902 | - | 1.180 | 1.013 | 0.843 | 1.322 | 1.194 | 1.078 | 1.306 | 0.813 | 0.783 | 1.444 | 1.237 | 1.189 | 1.211 | 1.204 | 1.059 |
| **P4** | 0.9827 | 1.163 | 0.935 | 1.116 | - | 0.944 | 1.114 | 1.179 | 1.136 | 1.253 | 1.089 | 0.842 | 0.891 | 1.325 | 1.511 | 1.010 | 0.988 | 0.831 | 1.217 |
| **P5** | 1.2197 | 0.946 | 0.968 | 1.65 | 1.01 | - | 1.302 | 1.426 | 1.763 | 1.381 | 1.215 | 0.929 | 1.249 | 1.463 | 1.736 | 1.027 | 1.074 | 1.048 | 1.126 |
| **P6** | 0.9831 | 0.867 | 0.787 | 1.479 | 0.869 | 0.909 | - | 0.913 | 1.997 | 0.869 | 1.136 | 0.955 | 0.978 | 1.170 | 1.440 | 1.101 | 1.002 | 1.319 | 0.924 |
| **P7** | 1.2472 | 1.163 | 1.019 | 1.400 | 0.903 | 1.402 | 0.791 | - | 1.184 | 1.233 | 1.271 | 0.986 | 1.088 | 1.578 | 1.276 | 1.004 | 1.210 | 1.063 | 1.052 |
| **P8** | 1.3979 | 1.137 | 0.683 | 1.112 | 0.807 | 0.891 | 0.957 | 0.960 | - | 0.859 | 0.928 | 0.813 | 1.027 | 1.304 | 1.334 | 0.969 | 1.292 | 1.094 | 1.063 |
| **P9** | 1.1401 | 0.868 | 0.92 | 1.685 | 1.103 | 0.902 | 1.199 | 1.318 | 1.404 | - | 1.039 | 0.892 | 1.153 | 1.355 | 1.588 | 1.292 | 1.431 | 1.168 | 1.224 |
| **P10** | 1.1704 | 0.838 | 1.335 | 1.534 | 1.285 | 0.925 | 0.761 | 1.068 | 1.36 | 1.296 | - | 0.845 | 1.040 | 1.173 | 1.517 | 0.978 | 1.267 | 1.650 | 0.979 |
| **P11** | 0.8986 | 0.752 | 0.906 | 1.125 | 0.876 | 1.091 | 0.980 | 1.007 | 1.575 | 0.991 | 1.524 | - | 1.117 | 1.188 | 1.291 | 0.846 | 1.048 | 1.423 | 1.314 |
| **P12** | 1.1076 | 1.173 | 1.000 | 1.461 | 1.085 | 1.097 | 0.641 | 1.354 | 1.192 | 1.244 | 1.164 | 0.822 | - | 1.135 | 1.665 | 0.769 | 1.556 | 1.131 | 1.043 |
| **P13** | 1.2899 | 0.868 | 1.01 | 1.217 | 0.837 | 0.805 | 0.877 | 1.232 | 1.302 | 0.806 | 0.999 | 0.843 | 0.88 | - | 0.932 | 0.953 | 1.404 | 1.089 | 1.140 |
| **P14** | 1.4377 | 1.06 | 0.738 | 1.039 | 1.17 | 0.657 | 1.034 | 1.068 | 1.887 | 0.947 | 1.086 | 0.943 | 0.894 | 1.396 | - | 1.071 | 1.193 | 1.144 | 1.089 |
| **P15** | 0.9679 | 1.131 | 0.953 | 1.239 | 1.053 | 0.963 | 0.919 | 1.026 | 1.15 | 1.161 | 1.074 | 1.108 | 0.867 | 1.358 | 1.005 | - | 1.044 | 0.965 | 0.998 |
| **P16** | 1.2439 | 1.13 | 0.664 | 1.114 | 1.011 | 1.130 | 0.951 | 1.236 | 1.383 | 1.02 | 1.302 | 0.845 | 0.88 | 1.322 | 1.701 | 0.917 | - | 1.042 | 1.176 |
| **P17** | 1.3674 | 1.151 | 1.109 | 1.455 | 0.935 | 0.995 | 1.219 | 0.927 | 1.731 | 0.839 | 0.829 | 0.797 | 0.574 | 1.319 | 1.098 | 0.907 | 1.328 | - | 1.184 |
| **P18** | 1.1597 | 0.999 | 0.937 | 1.252 | 0.814 | 0.914 | 0.934 | 1.130 | 1.76 | 0.951 | 1.266 | 1.005 | 0.782 | 1.339 | 1.407 | 0.761 | 1.293 | 1.317 | - |

**Supplementary Table S4 Recent gene-flow estimates (as rate of migration from population i into population j averaged from five computations) among 18 populations of *Carex moorcroftii* estimated by BAYESASS. Standard deviation is shown in parenthesis.**

| **Popi** | Pop j | | | | | | | | | | | | | | | | | |
| --- | --- | --- | --- | --- | --- | --- | --- | --- | --- | --- | --- | --- | --- | --- | --- | --- | --- | --- |
|  | North of TM | | | | | | | | | South of TM | | | | | | | | |
|  | P1 | P2 | P3 | P4 | P5 | P6 | P7 | P8 | P9 | P10 | P11 | P12 | P13 | P14 | P15 | P16 | P17 | P18 |
| **P1** | **0.8430**  **(0.0247)** | **0.0293**  **(0.0143)** | **0.0107**  **(0.0102)** | 0.0077  (0.0075) | 0.0077  (0.0074) | **0.0109**  **(0.0101)** | 0.0090  (0.0088) | 0.0080  (0.0080) | 0.0072  (0.0072) | 0.0077  (0.0074) | 0.0075  (0.0071) | 0.0075  (0.0072) | 0.0074  (0.0073) | 0.0076  (0.0072) | 0.0071  (0.0071) | 0.0075  (0.0073) | 0.0075  (0.0072) | 0.0070  (0.0067) |
| **P2** | 0.0071  (0.0070) | **0.6747**  **(0.0079)** | **0.0204**  **(0.0141)** | **0.0461**  **(0.0198)** | 0.0075  (0.0072) | **0.1375**  **(0.0294)** | **0.0275**  **(0.0169)** | 0.0075  (0.0072) | 0.0073  (0.0073) | 0.0071  (0.0069) | 0.0071  (0.0071) | 0.0072  (0.0071) | 0.0073  (0.0072) | 0.0072  (0.0071) | 0.0072  (0.0072) | 0.0070  (0.0068) | 0.0070  (0.0069) | 0.0073  (0.0071) |
| **P3** | 0.0065  (0.0064) | 0.0061  (0.0060) | **0.9014**  **(0.0198)** | 0.0066  (0.0065) | 0.0058  (0.0056) | 0.0066  (0.0065) | 0.0068  (0.0063) | 0.0054  (0.0053) | 0.0060  (0.0056) | 0.0058  (0.0053) | 0.0056  (0.0054) | 0.0058  (0.0059) | 0.0054  (0.0053) | 0.0054  (0.0052) | 0.0054  (0.0050) | 0.0052  (0.0052) | 0.0050  (0.0050) | 0.0054  (0.0053) |
| **P4** | 0.0073  (0.0073) | 0.0071  (0.0069) | 0.0069  (0.0069) | **0.8245**  **(0.0245)** | 0.0071  (0.0070) | **0.0628**  **(0.0195)** | 0.0067  (0.0066) | 0.0072  (0.0070) | 0.0067  (0.0067) | 0.0069  (0.0067) | 0.0070  (0.0071) | 0.0069  (0.0068) | 0.0071  (0.0072) | 0.0073  (0.0070) | 0.0070  (0.0071) | 0.0070  (0.0067) | 0.0071  (0.0069) | 0.0073  (0.0071) |
| **P5** | 0.0092  (0.0086) | 0.0069  (0.0068) | 0.0075  (0.0072) | 0.0071  (0.0070) | **0.6772**  **(0.0194)** | **0.1064**  **(0.0303)** | **0.0159**  **(0.0137)** | 0.0830  (0.0295) | 0.0071  (0.0070) | 0.0068  (0.0067) | 0.0073  (0.0072) | 0.0070  (0.0069) | 0.0091  (0.0084) | 0.0068  (0.0067) | **0.0224**  **(0.0252)** | 0.0069  (0.0067) | 0.0068  (0.0066) | 0.0068  (0.0066) |
| **P6** | 0.0075  (0.0072) | 0.0073  (0.0072) | 0.0088  (0.0086) | 0.0072  (0.0072) | 0.0092  (0.0089) | **0.8505**  **(0.0258)** | **0.0124**  **(0.0133)** | 0.0076  (0.0077) | 0.0074  (0.0074) | **0.0173**  **(0.0141)** | 0.0070  (0.0070) | 0.0085  (0.0085) | 0.0084  (0.0082) | 0.0078  (0.0076) | 0.0098  (0.0091) | 0.0084  (0.0080) | 0.0071  (0.0070) | 0.0077  (0.0076) |
| **P7** | 0.0070  (0.0069) | 0.0070  (0.0066) | **0.0156**  **(0.0110)** | 0.0060  (0.0058) | 0.0077  (0.0074) | **0.0335**  **(0.0349)** | **0.8523**  **(0.0394)** | 0.0069  (0.0067) | 0.0065  (0.0064) | 0.0065  (0.0066) | 0.0061  (0.0059) | 0.0087  (0.0083) | 0.0058  (0.0057) | 0.0063  (0.0062) | 0.0062  (0.0059) | 0.0058  (0.0054) | 0.0064  (0.0060) | 0.0057  (0.0055) |
| **P8** | 0.0052  (0.0050) | 0.0052  (0.0051) | **0.0101**  **(0.0072)** | 0.0050  (0.0051) | 0.0051  (0.0051) | **0.1642**  **(0.0948)** | 0.0054  (0.0054) | **0.7474**  **(0.0937)** | 0.0070  (0.0065) | 0.0049  (0.0049) | 0.0050  (0.0051) | 0.0051  (0.0050) | 0.0052  (0.0052) | 0.0049  (0.0050) | 0.0053  (0.0052) | 0.0051  (0.0049) | 0.0050  (0.0049) | 0.0049  (0.0048) |
| **P9** | 0.0072  (0.0070) | 0.0072  (0.0069) | **0.0110**  **(0.0089)** | 0.0064  (0.0063) | 0.0062  (0.0063) | **0.0188**  **(0.0112)** | **0.0402**  **(0.0153)** | 0.0065  (0.0063) | **0.8340**  **(0.0224)** | 0.0064  (0.0063) | 0.0064  (0.0062) | **0.0124**  **(0.0092)** | 0.0061  (0.0062) | 0.0064  (0.0062) | 0.0063  (0.0062) | 0.0062  (0.0060) | 0.0062  (0.0060) | 0.0063  (0.0062) |
| **P10** | 0.0055  (0.0055) | 0.0057  (0.0056) | 0.0060  (0.0060) | 0.0057  (0.0056) | 0.0056  (0.0055) | 0.0055  (0.0056) | 0.0058  (0.0055) | 0.0057  (0.0056) | 0.0057  (0.0055) | **0.8952**  **(0.0204)** | 0.0059  (0.0059) | **0.0120**  **(0.0081)** | 0.0062  (0.0061) | 0.0060  (0.0059) | 0.0057  (0.0058) | 0.0059  (0.0057) | 0.0060  (0.0060) | 0.0059  (0.0058) |
| **P11** | 0.0075  (0.0074) | 0.0074  (0.0072) | 0.0077  (0.0074) | 0.0080  (0.0078) | 0.0076  (0.0074) | 0.0077  (0.0075) | 0.0077  (0.0073) | 0.0077  (0.0075) | 0.0080  (0.0076) | 0.0077  (0.0080) | **0.8680**  **(0.0242)** | 0.0078  (0.0075) | 0.0079  (0.0077) | 0.0080  (0.0077) | 0.0079  (0.0080) | 0.0078  (0.0074) | 0.0078  (0.0076) | 0.0079  (0.0075) |
| **P12** | 0.0070  (0.0067) | 0.0084  (0.0079) | 0.0073  (0.0072) | 0.0068  (0.0067) | 0.0069  (0.0070) | 0.0084  (0.0076) | 0.0074  (0.0072) | **0.0321**  **(0.0145)** | 0.0079  (0.0077) | 0.0078  (0.0076) | 0.0073  (0.0073) | **0.8508**  **(0.0237)** | 0.0073  (0.0070) | 0.0069  (0.0068) | 0.0067  (0.0065) | 0.0069  (0.0067) | 0.0070  (0.0070) | 0.0070  (0.0070) |
| **P13** | 0.0050  (0.0048) | 0.0070  (0.0064) | 0.0051  (0.0052) | 0.0051  (0.0052) | 0.0062  (0.0063) | 0.0052  (0.0050) | 0.0094  (0.0076) | 0.0051  (0.0050) | 0.0049  (0.0049) | **0.0135**  **(0.0091)** | 0.0072  (0.0065) | 0.0055  (0.0056) | **0.8825**  **(0.0198)** | 0.0055  (0.0056) | 0.0053  (0.0051) | 0.0170  (0.0112) | 0.0056  (0.0054) | 0.0049  (0.0050) |
| **P14** | 0.0055  (0.0053) | 0.0057  (0.0055) | 0.0057  (0.0056) | 0.0056  (0.0055) | 0.0061  (0.0058) | 0.0066  (0.0064) | 0.0056  (0.0055) | 0.0057  (0.0054) | 0.0054  (0.0053) | 0.0722  (0.0195) | 0.0054  (0.0051) | 0.0056  (0.0055) | 0.0050  (0.0048) | **0.8026**  **(0.0220)** | **0.0193**  **(0.0107)** | 0.0067  (0.0061) | **0.0181**  **(0.0138)** | **0.0132**  **(0.0125)** |
| **P15** | 0.0072  (0.0069) | 0.0074  (0.0073) | 0.0076  (0.0074) | 0.0076  (0.0073) | 0.0075  (0.0073) | 0.0075  (0.0075) | 0.0076  (0.0074) | 0.0072  (0.0070) | 0.0071  (0.0069) | 0.0072  (0.0073) | 0.0091  (0.0085) | 0.0076  (0.0075) | 0.0072  (0.0075) | 0.0074  (0.0072) | **0.8728**  **(0.0238)** | 0.0073  (0.0073) | 0.0075  (0.0074) | 0.0073  (0.0073) |
| **P16** | 0.0113  (0.0082) | 0.0057  (0.0055) | 0.0058  (0.0058) | 0.0056  (0.0057) | 0.0058  (0.0058) | 0.0058  (0.0054) | 0.0058  (0.0057) | 0.0058  (0.0059) | 0.0054  (0.0052) | 0.0065  (0.0064) | 0.0056  (0.0056) | 0.0059  (0.0056) | 0.0061  (0.0061) | 0.0058  (0.0057) | 0.0056  (0.0058) | **0.8949**  **(0.0204)** | 0.0059  (0.0059) | 0.0068  (0.0064) |
| **P17** | 0.0059  (0.0059) | 0.0058  (0.0055) | 0.0057  (0.0056) | 0.0059  (0.0056) | 0.0055  (0.0055) | 0.0055  (0.0054) | 0.0059  (0.0056) | 0.0056  (0.0056) | 0.0056  (0.0058) | 0.0058  (0.0056) | 0.0056  (0.0056) | 0.0058  (0.0056) | 0.0060  (0.0058) | 0.0057  (0.0059) | 0.0060  (0.0056) | 0.0067  (0.0063) | **0.8268**  **(0.0879)** | **0.0803**  **(0.0882)** |
| **P18** | 0.0061  (0.0059) | 0.0064  (0.0066) | 0.0062  (0.0061) | 0.0062  (0.0059) | 0.0061  (0.0062) | 0.0064  (0.0062) | 0.0061  (0.0064) | 0.0060  (0.0062) | 0.0064  (0.0061) | 0.0062  (0.0062) | 0.0061  (0.0061) | 0.0064  (0.0062) | 0.0057  (0.0058) | 0.0063  (0.0061) | 0.0064  (0.0063) | 0.0078  (0.0075) | **0.0196**  **(0.0112)** | **0.8796**  **(0.0223)** |

**Supplementary Table S5 Descriptive statistics for 15 morphological traits of *Carex moorcroftii.* Abbreviations are the same as Table 2.**

| **Pop** | | ***L*_leaf_ (cm)** | ***W*_leaf_ (mm)** | ***N*_leaf_** | ***L*_rhizome_ (cm)** | ***H*_infructescence_ (cm)** | ***M*_rhizomes_ (g)** | ***M*_roots_ (g)** | ***M*_shoots_ (g)** | ***M*_infructescence_ (g)** | ***M*_1000seeds_ (g)** | ***N*_seeds_** | ***N*_spikelet_** | ***RSV*** | ***RAB*** | ***M*_total_**  **(g)** |
| --- | --- | --- | --- | --- | --- | --- | --- | --- | --- | --- | --- | --- | --- | --- | --- | --- |
| **P1** | Mean | 8.21 | 1.93 | 8.50 | 9.02 | 11.00 | 3.51 | 0.40 | 0.89 | 0.14 | 0.59 | 72.90 | 3.60 | 0.04 | 4.20 | 4.94 |
|  | SD | 1.50 | 0.38 | 2.07 | 2.08 | 0.90 | 2.22 | 0.26 | 0.74 | 0.05 | 0.03 | 11.06 | 0.84 | 0.03 | 0.90 | 3.21 |
|  | Min | 5.80 | 1.52 | 6.00 | 7.00 | 9.80 | 0.93 | 0.05 | 0.08 | 0.08 | 0.54 | 56.00 | 3.00 | 0.02 | 3.08 | 1.19 |
|  | Max | 10.48 | 2.85 | 12.00 | 14.00 | 12.60 | 7.46 | 0.82 | 2.47 | 0.21 | 0.63 | 87.00 | 5.00 | 0.10 | 5.51 | 10.96 |
| **P2** | Mean | 7.82 | 1.84 | 9.50 | 16.62 | 14.94 | 11.25 | 0.80 | 2.81 | 0.22 | 1.97 | 84.50 | 3.90 | 0.01 | 4.87 | 15.07 |
|  | SD | 1.03 | 0.29 | 3.50 | 3.39 | 2.56 | 1.75 | 0.32 | 1.32 | 0.14 | 0.27 | 22.62 | 0.99 | 0.01 | 2.39 | 3.37 |
|  | Min | 6.78 | 1.46 | 7.00 | 12.10 | 11.23 | 8.15 | 0.32 | 0.81 | 0.10 | 1.56 | 43.00 | 2.00 | 0.01 | 2.77 | 9.40 |
|  | Max | 10.10 | 2.43 | 19.00 | 22.10 | 19.00 | 13.91 | 1.33 | 5.09 | 0.48 | 2.40 | 112.00 | 5.00 | 0.04 | 9.27 | 20.74 |
| **P5** | Mean | 8.33 | 1.73 | 7.00 | 8.05 | 9.24 | 6.78 | 0.29 | 1.09 | 0.11 | 0.77 | 56.80 | 3.60 | 0.02 | 6.76 | 8.27 |
|  | SD | 0.77 | 0.16 | 1.05 | 1.28 | 1.34 | 4.45 | 0.15 | 1.01 | 0.02 | 0.12 | 8.63 | 0.70 | 0.01 | 1.45 | 5.57 |
|  | Min | 7.33 | 1.45 | 6.00 | 6.40 | 7.30 | 1.85 | 0.12 | 0.19 | 0.08 | 0.60 | 40.00 | 3.00 | 0.01 | 4.20 | 2.29 |
|  | Max | 9.42 | 2.03 | 9.00 | 9.90 | 11.80 | 15.66 | 0.56 | 3.18 | 0.15 | 0.92 | 69.00 | 5.00 | 0.04 | 8.48 | 19.52 |
| **P7** | Mean | 6.87 | 1.75 | 10.90 | 28.70 | 9.54 | 5.14 | 0.36 | 0.87 | 0.12 | 0.72 | 74.20 | 4.00 | 0.02 | 7.03 | 6.50 |
|  | SD | 1.20 | 0.38 | 3.84 | 6.25 | 2.09 | 1.08 | 0.18 | 0.60 | 0.11 | 0.10 | 10.02 | 1.05 | 0.02 | 3.08 | 1.77 |
|  | Min | 4.80 | 1.36 | 7.00 | 19.00 | 7.74 | 3.49 | 0.13 | 0.33 | 0.05 | 0.60 | 61.00 | 3.00 | 0.01 | 3.11 | 4.15 |
|  | Max | 9.35 | 2.30 | 17.00 | 42.00 | 14.00 | 6.78 | 0.58 | 2.14 | 0.42 | 0.84 | 97.00 | 6.00 | 0.06 | 12.58 | 9.41 |
| **P8** | Mean | 4.25 | 1.58 | 8.40 | 9.50 | 7.59 | 12.26 | 0.26 | 2.66 | 0.09 | 0.87 | 31.80 | 2.60 | 0.01 | 4.54 | 15.27 |
|  | SD | 0.67 | 0.11 | 0.97 | 1.94 | 1.16 | 9.03 | 0.18 | 2.06 | 0.04 | 0.06 | 6.11 | 0.70 | 0.02 | 0.87 | 11.25 |
|  | Min | 3.48 | 1.40 | 7.00 | 7.00 | 5.90 | 0.69 | 0.02 | 0.19 | 0.03 | 0.75 | 23.00 | 2.00 | 0.00 | 2.71 | 0.97 |
|  | Max | 5.66 | 1.74 | 10.00 | 13.00 | 9.90 | 28.27 | 0.59 | 6.40 | 0.14 | 0.97 | 41.00 | 4.00 | 0.07 | 5.98 | 35.28 |
| **P9** | Mean | 6.35 | 1.87 | 11.70 | 16.00 | 12.02 | 2.15 | 0.07 | 0.69 | 0.11 | 0.42 | 13.50 | 2.30 | 0.05 | 2.77 | 3.01 |
|  | SD | 1.09 | 0.26 | 2.50 | 2.00 | 1.74 | 1.29 | 0.09 | 0.41 | 0.05 | 0.06 | 2.72 | 0.67 | 0.03 | 0.62 | 1.76 |
|  | Min | 5.13 | 1.34 | 8.00 | 13.00 | 9.20 | 0.96 | 0.02 | 0.25 | 0.06 | 0.35 | 10.00 | 2.00 | 0.02 | 1.89 | 1.30 |
|  | Max | 8.30 | 2.24 | 16.00 | 19.00 | 14.50 | 5.04 | 0.32 | 1.63 | 0.20 | 0.51 | 18.00 | 4.00 | 0.11 | 3.77 | 7.11 |
| **P10** | Mean | 5.26 | 2.33 | 9.70 | 14.00 | 8.66 | 2.04 | 0.12 | 0.62 | 0.16 | 1.05 | 117.10 | 4.20 | 0.08 | 2.61 | 2.94 |
|  | SD | 1.35 | 0.42 | 1.83 | 1.49 | 1.62 | 1.77 | 0.05 | 0.43 | 0.15 | 0.11 | 25.60 | 0.42 | 0.07 | 0.89 | 2.22 |
|  | Min | 3.35 | 1.83 | 8.00 | 12.00 | 7.20 | 0.55 | 0.06 | 0.15 | 0.07 | 0.90 | 94.00 | 4.00 | 0.01 | 1.34 | 0.83 |
|  | Max | 7.53 | 2.93 | 14.00 | 16.00 | 12.50 | 5.98 | 0.22 | 1.46 | 0.43 | 1.24 | 166.00 | 5.00 | 0.27 | 4.04 | 7.74 |
| **P11** | Mean | 3.62 | 1.74 | 8.70 | 10.00 | 8.05 | 0.81 | 0.11 | 0.36 | 0.06 | 1.41 | 95.30 | 2.80 | 0.05 | 2.30 | 1.35 |
|  | SD | 0.42 | 0.22 | 1.64 | 1.35 | 1.40 | 0.39 | 0.04 | 0.18 | 0.07 | 0.07 | 20.48 | 0.42 | 0.04 | 0.53 | 0.60 |
|  | Min | 3.06 | 1.43 | 7.00 | 8.00 | 5.30 | 0.38 | 0.06 | 0.10 | 0.03 | 1.34 | 57.00 | 2.00 | 0.02 | 1.45 | 0.58 |
|  | Max | 4.24 | 2.03 | 12.00 | 12.00 | 10.00 | 1.60 | 0.17 | 0.75 | 0.27 | 1.52 | 128.00 | 3.00 | 0.16 | 3.22 | 2.56 |
| **P12** | Mean | 6.85 | 1.90 | 12.30 | 5.77 | 14.38 | 5.60 | 0.59 | 1.22 | 0.19 | 0.52 | 38.30 | 2.30 | 0.02 | 4.23 | 7.60 |
|  | SD | 0.89 | 0.29 | 1.57 | 2.26 | 3.03 | 2.92 | 0.29 | 0.39 | 0.17 | 0.06 | 10.04 | 0.48 | 0.01 | 0.81 | 3.55 |
|  | Min | 5.64 | 1.48 | 11.00 | 3.40 | 9.83 | 1.66 | 0.33 | 0.58 | 0.06 | 0.43 | 22.00 | 2.00 | 0.01 | 3.40 | 2.86 |
|  | Max | 7.78 | 2.42 | 16.00 | 11.00 | 20.00 | 10.54 | 1.22 | 1.91 | 0.65 | 0.61 | 62.00 | 3.00 | 0.05 | 5.42 | 14.16 |
| **P14** | Mean | 6.15 | 1.96 | 10.90 | 16.24 | 9.00 | 7.35 | 0.52 | 1.24 | 0.18 | 2.00 | 60.40 | 3.30 | 0.07 | 5.13 | 9.30 |
|  | SD | 1.92 | 0.30 | 3.18 | 1.49 | 0.94 | 10.67 | 0.53 | 1.78 | 0.20 | 0.15 | 19.49 | 0.67 | 0.12 | 1.81 | 13.07 |
|  | Min | 4.20 | 1.66 | 6.00 | 14.40 | 7.54 | 0.46 | 0.04 | 0.06 | 0.01 | 1.78 | 33.00 | 2.00 | 0.00 | 1.27 | 0.90 |
|  | Max | 10.60 | 2.58 | 15.00 | 19.00 | 10.50 | 36.13 | 1.68 | 5.98 | 0.62 | 2.22 | 93.00 | 4.00 | 0.37 | 6.84 | 44.41 |
| **P15** | Mean | 8.39 | 2.27 | 8.73 | 14.82 | - | 8.31 | 0.94 | 0.89 | - | - | - | - | - | 8.90 | 10.14 |
|  | SD | 1.67 | 0.16 | 2.37 | 3.76 | - | 7.88 | 0.94 | 0.61 | - | - | - | - | - | 2.75 | 9.34 |
|  | Min | 6.30 | 1.92 | 5.00 | 9.00 | - | 1.35 | 0.14 | 0.18 | - | - | - | - | - | 5.13 | 1.67 |
|  | Max | 11.06 | 2.53 | 12.00 | 19.25 | - | 25.80 | 3.42 | 2.20 | - | - | - | - | - | 13.28 | 31.42 |

**Supplementary Table S6 Estimates of slope where *b* is the allometric exponent and the intercept, where *a* is the allometric coefficient for** **log*M*_aboveground_- *logM*_belowground_, *logM*_shoots_-*logM*_infructescence_ and *logM*_roots_*-logM*_rhizomes_ regressions within populations of *Carex moorcroftii*.**

|  | **Pop code** | ***R*^2^** | **Slope (*b*)** | **95%CI**  **(*b*)** | **Intercept (*a*)** | **95%CI**  **(*a*)** | ***p*** | **vs. *b* = 1(H_0_)** |
| --- | --- | --- | --- | --- | --- | --- | --- | --- |
| **log*M*_aboveground_- *logM*_belowground_** | **P1** | 0.946 | 0.876 | 0.726~1.058 | 0.5988 | 0.539~0.659 | 0.145 | **=** |
|  | **P2** | 0.829 | 0.307 | 0.221~0.428 | 0.9441 | 0.894~0.994 | **0.000** | **<** |
|  | **P5** | 0.944 | 0.817 | 0.674~ 0.989 | 0.8095 | 0.757~0.862 | **0.040** | **<** |
|  | **P7** | 0.780 | 0.359 | 0.247~ 0.521 | 0.7582 | 0.722~0.795 | **0.000** | **<** |
|  | **P8** | 0.967 | 1.096 | 0.946~ 1.270 | 0.6211 | 0.543~0.699 | 0.190 | **=** |
|  | **P9** | 0.816 | 1.077 | 0.764~ 1.517 | 0.4433 | 0.350~0.537 | 0.639 | **=** |
|  | **P10** | 0.777 | 1.185 | 0.814~ 1.725 | 0.4253 | 0.281~0.570 | 0.337 | **=** |
|  | **P11** | 0.814 | 0.810 | 0.574~ 1.142 | 0.2717 | 0.138~0.406 | 0.200 | **=** |
|  | **P12** | 0.920 | 1.407 | 1.120~ 1.767 | 0.5685 | 0.508~0.629 | **0.008** | **>** |
|  | **P14** | 0.834 | 1.226 | 0.885~ 1.700 | 0.6871 | 0.514~0.861 | 0.192 | **=** |
|  | **P15** | 0.955 | 1.340 | 1.143~ 1.570 | 0.9817 | 0.909~1.054 | **0.002** | **>** |
| ***logM*_shoots_-*logM*_infructescence_** | **P1** | 0.541 | 0.340 | 0.201~0.577 | -0.8145 | -0.910~-0.720 | **0.001** | **<** |
|  | **P2** | 0.236 | 0.991 | 0.511~1.924 | -1.1282 | -1.464~-0.793 | 0.978 | **=** |
|  | **P5** | 0.356 | 0.211 | 0.114~0.390 | -0.9584 | -1.017~-0.900 | **0.000** | **<** |
|  | **P7** | 0.169 | 1.011 | 0.508~2.011 | -0.8635 | -1.119~-0.608 | 0.974 | **=** |
|  | **P8** | 0.003 | 0.535 | 0.255~1.126 | -1.2607 | -1.534~-0.988 | 0.096 | **=** |
|  | **P9** | 0.018 | 0.766 | 0.366~ 1.604 | -0.8196 | -1.049~-0.590 | 0.464 | **=** |
|  | **P10** | 0.006 | 0.972 | 0.462~2.042 | -0.6115 | -1.012~-0.212 | 0.937 | **=** |
|  | **P11** | 0.093 | 1.165 | 0.570~2.380 | -0.7445 | -1.255~-0.234 | 0.661 | **=** |
|  | **P12** | 0.266 | 1.817 | 0.947~3.485 | -0.9314 | -1.138~-0.725 | 0.070 | **=** |
|  | **P14** | 0.001 | -0.938 | -1.974~-0.446 | -1.2732 | -1.886~-0.661 | 0.860 | **=** |
| ***logM*_roots_*-logM*_rhizomes_** | **P1** | 0.913 | 0.811 | 0.639~1.029 | 0.8761 | 0.751~1.001 | 0.077 | **=** |
|  | **P2** | 0.960 | 0.375 | 0.319~0.442 | 1.0952 | 1.082~1.108 | **0.000** | **<** |
|  | **P5** | 0.882 | 1.252 | 0.949~1.651 | 1.4875 | 1.265~1.710 | 0.099 | **=** |
|  | **P7** | 0.438 | 0.355 | 0.199~0.632 | 0.8841 | 0.759~1.009 | **0.002** | **<** |
|  | **P8** | 0.913 | 1.108 | 0.873~1.407 | 1.7212 | 1.504~1.939 | 0.355 | **=** |
|  | **P9** | 0.737 | 0.687 | 0.458~1.032 | 1.1821 | 0.791~1.573 | 0.067 | **=** |
|  | **P10** | 0.774 | 1.960 | 1.342~2.862 | 2.0512 | 1.315~2.788 | **0.003** | **˃** |
|  | **P11** | 0.100 | 1.174 | 0.576~2.391 | 1.0182 | 0.112~1.925 | 0.644 | **˃** |
|  | **P12** | 0.140 | 1.234 | 0.614~2.480 | 1.0220 | 0.706~1.338 | 0.537 | **=** |
|  | **P14** | 0.929 | 1.026 | 0.826~1.273 | 1.0960 | 0.933~1.259 | 0.796 | **=** |
|  | **P15** | 0.881 | 1.005 | 0.777~1.299 | 0.9433 | 0.823~1.064 | 0.969 | **=** |

**Supplementary Table S7 Results of mantel tests between pairwise differentiation in morphological traits, genetic distance, environmental distance and partial mantel tests controlling for pairwise differentiation in neutral genetic markers (*GD*) or geographic distance (GGD).**

| **Factors** |  | **GD** | | ***L*_leaf_** | ***W*_leaf_** | ***N*_leaf_** | ***L*_rhizome_** | ***H*_infructescence_** | ***M*_rhizome_** | ***M*_roots_** | ***M*_shoots_** | ***M*_infructescence_** | ***M*_1000seeds_** | ***N*_seeds_** | ***N*_spikelet_** | ***RSV*** | ***RAB*** | ***M*_total_** |
| --- | --- | --- | --- | --- | --- | --- | --- | --- | --- | --- | --- | --- | --- | --- | --- | --- | --- | --- |
| **Bio1** | r | **0.465** | | -0.080 | 0.303 | -0.188 | **-0.197** | -0.104 | -0.096 | **0.516** | -0.161 | -0.002 | 0.315 | -0.228 | **-0.212** | 0.085 | 0.435 | -0.102 |
|  | p | **0.002** | | 0.419 | 0.152 | 0.080 | **0.044** | 0.392 | 0.385 | **0.016** | 0.289 | 0.621 | 0.096 | 0.067 | **0.041** | 0.248 | 0.062 | 0.390 |
| **Bio4** | r | **0.315** | | -0.044 | 0.274 | **-0.240** | -0.113 | -0.217 | -0.102 | 0.394 | -0.180 | -0.177 | 0.040 | **-0.321** | -0.151 | 0.084 | 0.417 | -0.113 |
|  | p | **0.034** | | 0.468 | 0.177 | **0.045** | 0.465 | 0.121 | 0.357 | 0.085 | 0.340 | 0.199 | 0.397 | **0.014** | 0.142 | 0.252 | 0.079 | 0.337 |
| **Bio5** | r | **0.377** | | -0.055 | 0.249 | -0.165 | **-0.228** | **-0.238** | -0.124 | **0.458** | -0.204 | -0.175 | 0.081 | -0.124 | -0.158 | 0.009 | 0.393 | -0.130 |
|  | p | **0.005** | | 0.400 | 0.138 | 0.124 | **0.012** | **0.044** | 0.256 | **0.028** | 0.119 | 0.171 | 0.278 | 0.309 | 0.114 | 0.396 | 0.063 | 0.268 |
| **Bio6** | r | **0.415** | | -0.027 | 0.365 | -0.181 | **-0.192** | -0.139 | -0.074 | **0.548** | -0.174 | -0.057 | 0.299 | -0.096 | -0.136 | 0.124 | 0.156 | -0.088 |
|  | p | **0.006** | | 0.565 | 0.143 | 0.118 | **0.033** | 0.298 | 0.510 | **0.015** | 0.238 | 0.463 | 0.125 | 0.379 | 0.194 | 0.226 | 0.174 | 0.458 |
| **Bio9** | r | **0.518** | | -0.090 | 0.297 | -0.178 | -0.184 | -0.055 | -0.075 | **0.515** | -0.147 | 0.078 | **0.421** | -0.225 | -0.153 | 0.165 | 0.434 | -0.082 |
|  | p | **0.002** | | 0.353 | 0.154 | 0.156 | 0.140 | 0.529 | 0.444 | **0.018** | 0.370 | 0.275 | **0.043** | 0.140 | 0.166 | 0.177 | 0.091 | 0.430 |
| **Bio10** | r | **0.392** | | -0.064 | 0.269 | -0.177 | **-0.218** | -0.201 | -0.121 | **0.483** | -0.191 | -0.115 | 0.157 | -0.184 | -0.187 | 0.020 | 0.407 | -0.126 |
|  | p | **0.004** | | 0.459 | 0.167 | 0.125 | **0.011** | 0.091 | 0.331 | **0.036** | 0.127 | 0.311 | 0.164 | 0.173 | 0.062 | 0.366 | 0.066 | 0.287 |
| **Bio11** | r | **0.486** | | -0.084 | 0.310 | -0.174 | -0.187 | -0.029 | -0.074 | **0.536** | -0.141 | 0.093 | **0.401** | -0.221 | -0.155 | 0.139 | 0.455 | -0.080 |
|  | p | **0.004** | | 0.374 | 0.145 | 0.160 | 0.117 | 0.591 | 0.459 | **0.015** | 0.398 | 0.264 | **0.049** | 0.150 | 0.168 | 0.202 | 0.079 | 0.449 |
| **Bio12** | r | 0.084 | | 0.024 | -0.232 | -0.087 | -0.130 | -0.028 | -0.069 | -0.107 | 0.020 | -0.033 | 0.075 | **-0.265** | -0.056 | 0.096 | -0.210 | -0.043 |
|  | p | 0.276 | | 0.366 | 0.103 | 0.334 | 0.347 | 0.516 | 0.395 | 0.354 | 0.334 | 0.544 | 0.299 | **0.039** | 0.399 | 0.257 | 0.111 | 0.486 |
| **GGD** | r | **0.327** | | 0.046 | 0.073 | -0.132 | -0.153 | 0.001 | -0.078 | 0.233 | -0.013 | 0.004 | 0.078 | **-0.250** | 0.020 | 0.134 | 0.145 | -0.055 |
|  | p | **0.022** | | 0.352 | 0.274 | 0.247 | 0.250 | 0.447 | 0.305 | 0.075 | 0.533 | 0.416 | 0.276 | **0.026** | 0.370 | 0.165 | 0.221 | 0.412 |
| **GD** | r | - | | 0.009 | **0.423** | **-0.263** | -0.204 | -0.054 | 0.061 | 0.216 | 0.037 | 0.246 | 0.017 | 0.189 | -0.016 | **0.435** | 0.208 | 0.074 |
|  | p | - | | 0.430 | **0.023** | **0.036** | 0.135 | 0.444 | 0.334 | 0.112 | 0.355 | 0.109 | 0.434 | 0.189 | 0.501 | **0.015** | 0.127 | 0.308 |
|  |  | | **Controlling for GGD** | | | | | | | | | | | | | | | |
| **Bio1** | r | **0.382** | | -0.110 | 0.301 | -0.146 | -0.147 | -0.105 | -0.069 | **0.473** | -0.172 | -0.002 | 0.307 | -0.196 | **-0.217** | 0.065 | 0.416 | -0.086 |
|  | p | **0.008** | | 0.291 | 0.118 | 0.192 | 0.220 | 0.391 | 0.415 | **0.007** | 0.173 | 0.378 | 0.091 | 0.109 | **0.038** | 0.266 | 0.053 | 0.357 |
| **Bio4** | r | 0.101 | | -0.107 | 0.315 | -0.208 | -0.007 | **-0.306** | -0.066 | 0.333 | -0.241 | -0.253 | -0.021 | -0.211 | -0.232 | -0.013 | **0.448** | -0.104 |
|  | p | 0.165 | | 0.279 | 0.131 | 0.086 | 0.419 | **0.022** | 0.395 | 0.056 | 0.073 | 0.067 | 0.473 | 0.124 | 0.051 | 0.461 | **0.046** | 0.298 |
| **Bio5** | r | **0.295** | | -0.074 | 0.239 | -0.128 | -0.189 | **-0.240** | -0.104 | **0.414** | -0.211 | -0.176 | 0.073 | -0.101 | -0.160 | -0.005 | **0.369** | -0.118 |
|  | p | **0.027** | | 0.362 | 0.143 | 0.250 | 0.087 | **0.034** | 0.289 | **0.013** | 0.095 | 0.157 | 0.264 | 0.340 | 0.091 | 0.482 | **0.045** | 0.247 |
| **Bio6** | r | **0.296** | | -0.07 | 0.055 | -0.121 | 0.280 | -0.140 | -0.132 | 0.039 | -0.177 | -0.057 | 0.293 | -0.073 | -0.138 | 0.112 | **0.444** | -0.148 |
|  | p | **0.038** | | 0.363 | 0.373 | 0.248 | 0.064 | 0.295 | 0.230 | 0.376 | 0.121 | 0.494 | 0.097 | 0.409 | 0.188 | 0.225 | **0.015** | 0.188 |
| **Bio9** | r | **0.427** | | 0.044 | 0.298 | -0.132 | -0.128 | -0.056 | -0.043 | **0.472** | -0.159 | 0.079 | **0.415** | -0.178 | -0.162 | 0.139 | **0.418** | -0.062 |
|  | p | **0.004** | | 0.357 | 0.138 | 0.254 | 0.295 | 0.500 | 0.433 | **0.020** | 0.246 | 0.245 | **0.034** | 0.197 | 0.149 | 0.192 | **0.049** | 0.412 |
| **Bio10** | r | **0.326** | | -0.088 | 0.261 | -0.138 | -0.174 | -0.203 | -0.099 | **0.438** | -0.200 | -0.116 | 0.148 | -0.156 | -0.191 | 0.002 | **0.384** | -0.113 |
|  | p | **0.014** | | 0.350 | 0.140 | 0.203 | 0.131 | 0.081 | 0.325 | **0.014** | 0.109 | 0.268 | 0.159 | 0.189 | 0.057 | 0.417 | **0.028** | 0.265 |
| **Bio11** | r | **0.384** | | -0.119 | 0.312 | -0.127 | -0.131 | -0.030 | -0.042 | **0.497** | -0.152 | 0.094 | **0.394** | -0.175 | -0.164 | 0.112 | **0.442** | -0.061 |
|  | p | **0.004** | | 0.255 | 0.132 | 0.265 | 0.276 | 0.416 | 0.477 | **0.009** | 0.223 | 0.240 | **0.048** | 0.198 | 0.130 | 0.194 | **0.037** | 0.449 |
| **Bio12** | r | -0.130 | | -0.014 | **-0.421** | 0.015 | -0.026 | -0.081 | -0.017 | **-0.419** | 0.042 | -0.104 | 0.005 | -0.092 | -0.218 | -0.085 | **-0.470** | -0.004 |
|  | p | 0.261 | | 0.449 | **0.050** | 0.499 | 0.398 | 0.435 | 0.391 | **0.006** | 0.401 | 0.341 | 0.372 | 0.399 | 0.068 | 0.347 | **0.015** | 0.462 |
| **GD** | r | - | | -0.007 | **0.425** | -0.234 | -0.161 | -0.054 | 0.095 | 0.147 | 0.044 | 0.246 | 0.009 | 0.222 | -0.017 | **0.428** | 0.169 | 0.099 |
|  | p | - | | 0.495 | **0.025** | 0.091 | 0.198 | 0.431 | 0.262 | 0.187 | 0.402 | 0.114 | 0.452 | 0.144 | 0.490 | **0.020** | 0.175 | 0.256 |
|  |  | | **Controlling for GD** | | | | | | | | | | | | | | | |
| **Bio1** | r | - | | -0.095 | 0.136 | -0.078 | -0.120 | -0.098 | -0.043 | **0.480** | -0.200 | -0.035 | 0.315 | **-0.258** | **-0.211** | 0.033 | **0.390** | -0.152 |
|  | p | - | | 0.317 | 0.245 | 0.360 | 0.257 | 0.401 | 0.433 | **0.019** | 0.141 | 0.498 | 0.091 | **0.037** | **0.050** | 0.361 | **0.036** | 0.203 |
| **Bio4** | r | - | | -0.052 | 0.114 | -0.145 | -0.029 | -0.217 | -0.142 | 0.342 | -0.216 | -0.187 | 0.039 | **-0.330** | -0.150 | 0.085 | 0.370 | -0.160 |
|  | p | - | | 0.480 | 0.321 | 0.243 | 0.414 | 0.131 | 0.233 | 0.058 | 0.175 | 0.162 | 0.353 | **0.010** | 0.160 | 0.290 | 0.065 | 0.244 |
| **Bio5** | r | - | | -0.053 | 0.138 | -0.091 | -0.177 | **-0.242** | -0.150 | **0.421** | -0.226 | -0.165 | 0.082 | -0.115 | -0.158 | 0.038 | **0.353** | -0.160 |
|  | p | - | | 0.431 | 0.213 | 0.326 | 0.137 | **0.041** | 0.163 | **0.022** | 0.083 | 0.145 | 0.291 | 0.290 | 0.100 | 0.340 | **0.046** | 0.162 |
| **Bio6** | r | - | | 0.052 | -0.082 | -0.062 | **0.333** | -0.134 | -0.174 | 0.013 | **-0.200** | -0.090 | 0.298 | -0.122 | -0.134 | 0.078 | **0.423** | -0.191 |
|  | p | - | | 0.324 | 0.349 | 0.395 | **0.037** | 0.303 | 0.091 | 0.422 | **0.017** | 0.373 | 0.108 | 0.324 | 0.170 | 0.290 | **0.015** | 0.052 |
| **Bio9** | r | - | | -0.110 | 0.109 | -0.055 | -0.097 | -0.043 | -0.122 | **0.481** | -0.191 | 0.024 | 0.428 | **-0.279** | -0.153 | 0.076 | **0.389** | -0.137 |
|  | p | - | | 0.312 | 0.260 | 0.443 | 0.341 | 0.482 | 0.265 | **0.021** | 0.203 | 0.366 | 0.041 | **0.035** | 0.166 | 0.298 | **0.047** | 0.252 |
| **Bio10** | r | - | | -0.072 | 0.136 | -0.090 | -0.157 | -0.201 | -0.154 | 0.444 | -0.219 | -0.117 | 0.157 | -0.186 | **-0.186** | 0.024 | **0.363** | -0.164 |
|  | p | - | | 0.387 | 0.247 | 0.319 | 0.201 | 0.101 | 0.174 | 0.025 | 0.097 | 0.284 | 0.167 | 0.154 | **0.042** | 0.399 | **0.038** | 0.146 |
| **Bio11** | r | - | | -0.102 | 0.126 | -0.050 | -0.100 | -0.018 | -0.120 | 0.506 | -0.183 | -0.100 | **0.406** | -0.271 | -0.155 | 0.053 | **0.413** | -0.135 |
|  | p | - | | 0.348 | 0.270 | 0.438 | 0.319 | 0.401 | 0.289 | 0.013 | 0.193 | 0.319 | **0.044** | 0.051 | 0.142 | 0.347 | **0.041** | 0.238 |
| **Bio12** | r | - | | 0.024 | -0.255 | -0.090 | -0.132 | -0.025 | -0.069 | -0.109 | 0.019 | -0.041 | 0.074 | **-0.275** | -0.056 | 0.092 | -0.214 | -0.043 |
|  | p | - | | 0.385 | 0.069 | 0.336 | 0.323 | 0.477 | 0.417 | 0.310 | 0.335 | 0.476 | 0.304 | **0.021** | 0.399 | 0.276 | 0.087 | 0.446 |
| **GGD** | r | - | | 0.045 | -0.090 | -0.043 | -0.088 | 0.005 | -0.106 | 0.171 | -0.027 | -0.020 | 0.076 | **-0.274** | 0.021 | 0.102 | 0.078 | -0.087 |
|  | p | - | | 0.349 | 0.336 | 0.424 | 0.416 | 0.437 | 0.296 | 0.137 | 0.483 | 0.499 | 0.248 | **0.017** | 0.331 | 0.218 | 0.323 | 0.289 |

Notes: Bio1, Annual Mean Temperature; Bio 4, Temperature Seasonality (standard deviation *100); Bio 5, Max Temperature of Warmest Month; Bio 6, Min Temperature of Coldest Month; Bio 9, Mean Temperature of Driest Quarter; Bio 10, Mean Temperature of Warmest Quarter; Bio 11, Mean Temperature of Coldest Quarter; Bio 12, Annual Precipitation.

**Supplementary Fig. S1 Relative importance of the 19 bioclimatic variables in explaining the distribution of *C. moorcroftii*.** The percentage contribution indicates the percentage of variance explained by that particular variable. The permutation importance test indicates the relative importance of bioclimatic variables. (Abbreviations: P., precipitation; Tem., temperature).

**Supplementary Fig. S2 The results of the jackknife test of 19 climate variables’ contribution in modelling *Carex moorcroftii*’s habitat distribution.** (The regularized training gain describes how much the Maxent distribution fits the presence data compared to a uniform distribution.

**Supplementary Fig. S3 Principal-Coordinate-Analysis (PCoA) of the 19 climatic variables of 18 sample points.**

**Supplementary Fig. S4** Scatter plots of ΔK showing the difference between consecutive log-transformed probabilities (ΔK) of the data when K increases.

**Supplementary Fig. S5** Principal-Coordinate-Analysis (PCoA) of the 15 morphological traits at different sample points.

**
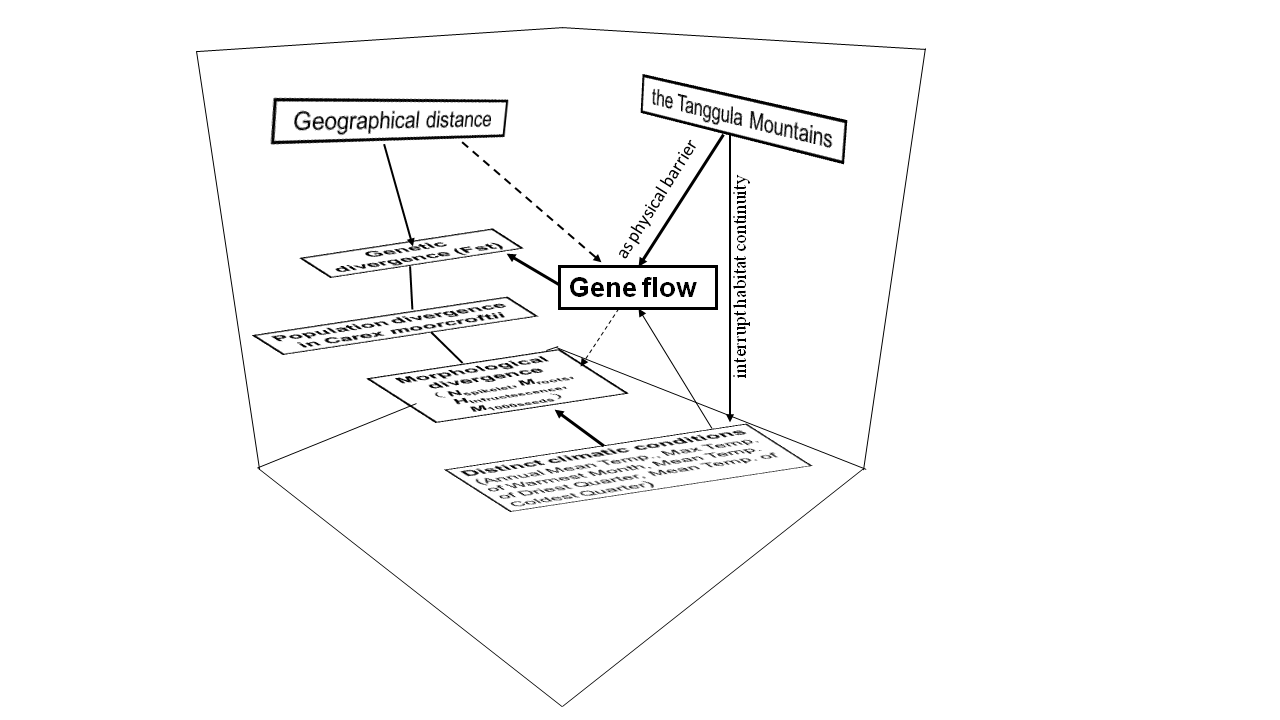
**

**Supplementary Fig. S6 Diagram of the effects of geographic distance, physical barrier, bioclimatic variables on genetic distance and morphological divergence. Solid line indicate direct effect, Dashed arrows indicate indirect effect, Line weight show the strength.**
